# Machine Learning‐Guided Engineering of Protein Phase Separation Properties in Immune Regulation

**DOI:** 10.1002/advs.202520890

**Published:** 2026-02-11

**Authors:** Chenqiu Zhang, Jia Wang, Zhe Wang, Liyan Zhu, Sihui Cai, Luzhi Zhan, Haorui Liang, Yaoxing Wu, Jianqiang Li, Jun Cui

**Affiliations:** ^1^ MOE Key Laboratory of Gene Function and Regulation Guangdong Province Key Laboratory of Pharmaceutical Functional Genes State Key Laboratory of Biocontrol, Innovation Center of the Sixth Affiliated Hospital, Center of Evolutionary Synthetic Biology School of Life Sciences Sun Yat‐sen University Guangzhou Guangdong China; ^2^ College of Computer Science and Software Engineering Shenzhen University Shenzhen China; ^3^ Department of Critical Care Medicine, Institute of Precision Medicine The First Affiliated Hospital of Sun Yat‐sen University Sun Yat‐sen University Guangzhou Guangdong China; ^4^ School of Artificial Intelligence, National Engineering Laboratory for Big Data System Computing Technology Shenzhen University Shenzhen Guangdong China

**Keywords:** cGAS, contractive learning, innate immunity, machine learning, protein phase separation

## Abstract

Phase separation (PS) underpins compartmentalization in living cells, facilitating the formation of membraneless organelles and the regulation of cellular processes. Despite the increasingly pivotal role of engineering protein PS properties in the study and regulation of cellular physiological processes, manipulating PS ability through single amino acid alterations remains a challenge. Here, we develop phase separation scalpel (PScalpel), a machine learning‐based tool identifying and recommends protein engineering strategies for directed changes in PS ability. Based on our biological experimental data, we apply transfer learning to achieve the feedback‐driven optimization of specific protein prediction accuracy–‐markedly enhancing the predictive performance for TDP43, a neurodegenerative disease‐associated protein. Furthermore, by engineering the crucial nucleic acid sensor cGAS as a model application, we successfully modulate its PS ability in the anticipated direction by altering a single amino acid, which subsequently optimizes its immune function and impacts the activity of engineered macrophages. Transcriptomic analysis of these cGAS‐engineered macrophages further demonstrated that the immune function of macrophages can be altered by the manipulation of cGAS PS ability. In summary, PScalpel is an effective tool for guiding PS ability engineering, enabling targeted molecular and cellular modifications and providing nuanced methods for precise biomolecular engineering in future research.

## Graphical abstract

PScalpel, a machine learning model integrating protein structure extraction, graph contrastive learning, and a genetic algorithm, guides the engineering of protein phase separation ability. It adopts transfer learning methods to provide predictive recommendations for protein phase separation ability changes through single amino acid mutations in a feedback‐driven manner, enabling accurate immune regulation via cGAS engineering in macrophages.

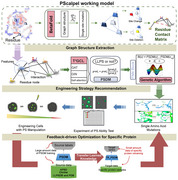

## 1 Introduction

Protein phase separation (PS), especially liquid‒liquid PS (LLPS), is a critical biological process that facilitates the formation of membraneless organelles, concentrating biochemical reactants to optimize cellular functions [1, 2]. The PS process is closely connected with various diseases, including neurodegenerative diseases [3, 4], cancer [5, 6] and pathogen‐induced hyperinflammation [4, 7]. Mutations in certain proteins, such as FUS [8, 9], TDP43 [4, 10, 11] and tau [12, 13], are known to induce dysfunctional PS, leading to compromised protein functionality. The driving forces of protein PS include multivalency interactions, oligomerization of structural domains, and the interplay of structural domains with low‐complexity sequences [1, 14, 15, 16, 17, 18]. Alterations in specific amino acids can perturb these interactions, thus disrupting the PS properties of proteins. It has great value for meeting the rapidly expanding need of therapeutic and medical synthetic biology via adjustable PS ability, since it can quickly impact protein function, thus affecting cellular biological processes. Consequently, there is a growing need for advanced computational tools to predict and guide amino acid transitions to regulate protein PS ability [19, 20, 21, 22, 23].

In response to the burgeoning demand for bioinformatics tools capable of predicting and validating the PS properties of target proteins, sophisticated tools such as PhaSePred [24], PScore [25], catGRANULE [26], PSPredictor [27], DeePhase [28] and Phase Separation Analysis and Prediction (PSAP) [29] have been developed. These tools, particularly PhaSePred, PSPredictor, DeePhase, and PSAP, represent significant advancements as second‐generation tools, employing machine learning algorithms trained on diverse protein features to identify PS‐related sequences with high accuracy. Since most of these tools are designed to predict natural proteins with substantial sequence differences rather than protein mutants, they lack the ability to detect subtle changes in PS propensity induced by single amino acid mutations. This highlights the need for tools to guide the alteration of protein PS capabilities.

To this end, we developed phase separation scalpel (PScalpel), a machine learning‐based tool, for predicting alterations in protein PS behavior. PScalpel consists of a novel module of BetaFold, which was built upon the foundation of Alphafold2 and was intended to provide an efficient and accurate tool for extracting structural information. Twin tower graph contrastive learning (T^3^GCL), developed from GCL, enhances the overall performance and sensitivity of the model, integrates a genetic algorithm (GA) to accelerate the search for optimal alterations, and incorporates transfer learning (TL) to improve the accuracy in specific protein. On the basis of these modules, we overcame the complex challenge of utilizing protein three‐dimensional (3D) structure information and verified its universality and feasibility by biological experimental methods.

As the most important DNA recognition receptor in innate immunity, cGAS can respond effectively to various DNA release events, activating the cGAS‐STING signaling pathway to exert multifunctional immune responses against viral infection and tumour progression [30]. The cGAS‐DNA complex is pivotal in driving PS, which is essential for efficient DNA sensing and resistance to negative regulation [30, 31]. PS mutations in cGAS influence its activity through condensation abrogation [30, 32]. Thus, modulating the PS ability of cGAS enabled specific interventions in its immune functions, facilitating precise cellular engineering. In this study, we utilized the PScalpel to manipulate the PS function of cGAS and engineered macrophage functions based on these findings, thereby circumventing the need for extensive experimental trial‐and‐error. Through single amino acid modification, we are able to minimize disruptions to the native structure and function while eliciting the anticipated changes in protein PS properties. Importantly, this tool and application strategy hold significant potential for broad application to other proteins, facilitating the precise and targeted modulation of molecular and cellular processes.

## 2 Results

### 2.1 Establishment of PScalpel to Guide the Alteration of PS Ability

As the existing PS prediction tools, such as PSAP [29] have been developed for natural proteins, there are no suitable tools to distinguish the PS ability of mutated proteins. We developed PScalpel to precisely discriminate the PS likelihood of mutated proteins and were capable of screening out the optimal mutation strategy that meets the demand. By employing a graphical contrastive learning methodology, PScalpel was able to discern pivotal amino acids that are critical for protein PS, and tailored modification strategies were proposed. PScalpel utilized well‐annotated data and protein 3D structure information from Liquid‐liquid Phase Separation Database (LLPSDB) [33], National Center for Biotechnology Information (NCBI) [34] and Protein Data Bank (PDB) [35], which contain more information than 2D sequences do. Thus, detailed structural data incorporating machine learning support a high‐sensitivity model to detect subtle differences between mutations with increased accuracy (Figure S1A and Data S1).

**FIGURE 1:**
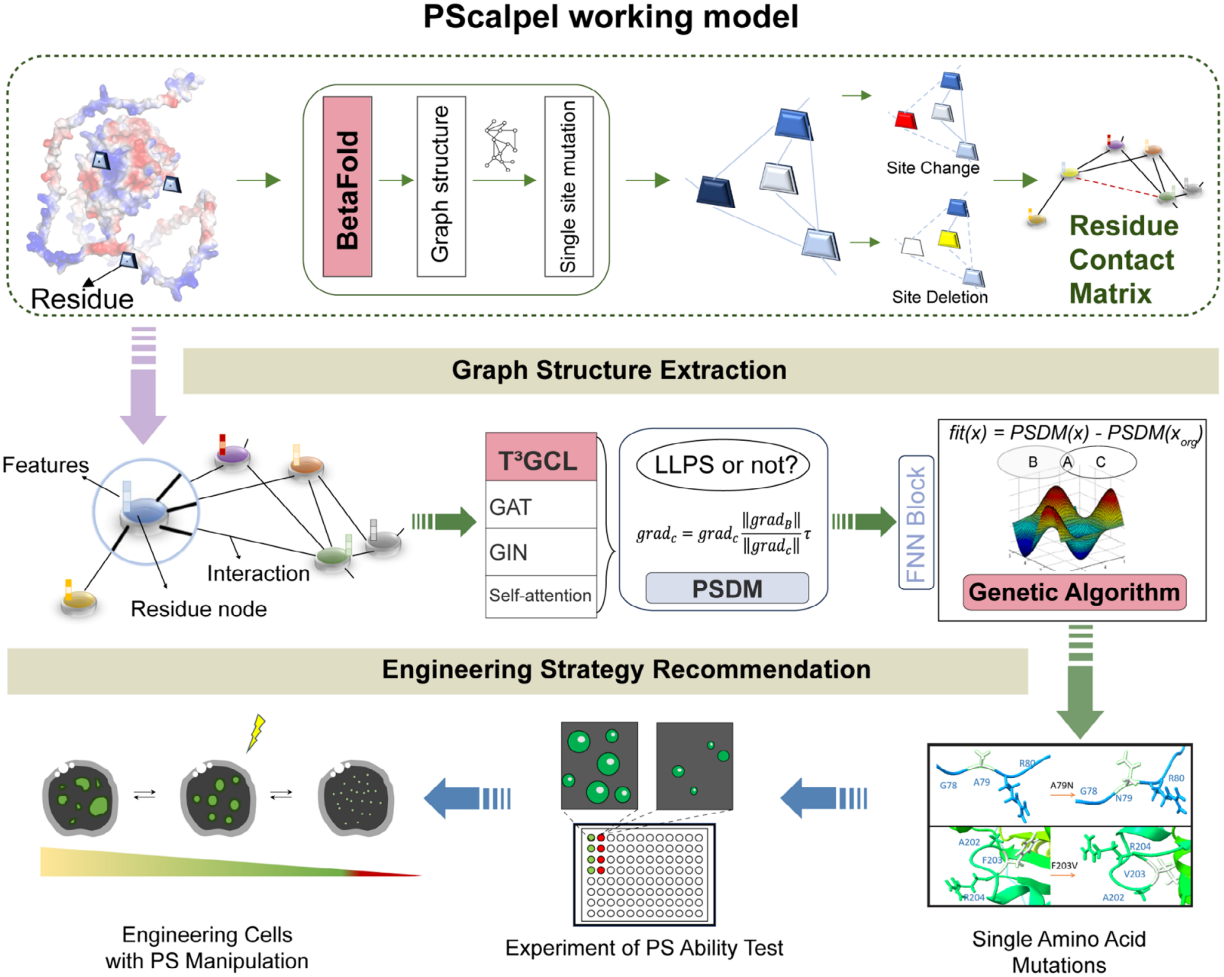
Establishment of PScalpel to guide the alteration of PS ability. Schematic diagram showing the working models of PScalpel. PScalpel was composed in a hierarchical manner. It took protein sequence as input, then obtaining the residue interaction relationship based on graph structure extracted by BetaFold module. Distinguishing the PS ability of mutated proteins under the joint action of PSDM module. GA was responsible for strategy recommendation. Then, the association between genotype and phenotype was verified through experiments, enabling the construction of engineered cells.

PScalpel comprises three core components that work in tandem: BetaFold module for graph structure extraction, T^3^GCL module for PS prediction, and a GA module for engineering strategy recommendation (Figure 1).

The core module, BetaFold, was designed to extract protein graph structures by analyzing residue sites (including variations and deletions) and converting them into a residue contact matrix (RCM). Building on AlphaFold2's Transformer framework, BetaFold adopted a lightweight variant that efficiently captured long‐range residue associations while retaining structural priors. We sourced 23 391 human protein sequences from the AlphaFold Protein Structure Database (APSD) for training and validation, and 731 experimental native protein structures from the RCSB‐PDB database for testing (Table S1 and Figure S1B). A critical optimization replaced computationally costly multiple sequence alignment (MSA) pipelines with AlphaFold2‐predicted structures as training pseudo‐labels, cutting down computational expense and simplifying the task to binary classification—judging if amino acid pairs are adjacent, defined as C_α_ atoms within 10 Å, instead of predicting full atomic coordinates (Figure S1C). Combining this pseudo‐label strategy with the improved lightweight Transformer, BetaFold efficiently generated residue‐level contact graphs, preserved structural priors, and balanced computational efficiency, prediction accuracy, and freedom from expensive preprocessing.

The intermediate module T^3^GCL is a custom graph convolutional network (GCN) developed to generate positive pairs for contrastive learning without reducing model sensitivity. When fed with RCM and labeled protein features, T^3^GCL uses a target dataset plus two auxiliary datasets to learn consistent protein information. The first auxiliary task studies mutation‐driven functional changes with benign and pathogenic mutant proteins from ClinVar, while the second identifies PS features with PS‐capable proteins from LLPSDB and non‐PS proteins from PDB (Table S2, Figure S1D,E). Integrated with self‐attention [36], graph isomorphism (GIN) [37, 38], and graph attention (GAT) [39] (Figure S1F), T^3^GCL enhances the capture of local and global protein structural patterns, boosting PS prediction accuracy. Combined with BetaFold, T^3^GCL forms the PSDM, which effectively distinguishes the PS ability of mutant proteins using extracted structural data and consistent features from auxiliary tasks.

The outer module, GA, guides targeted protein engineering. Its fitness function is defined as fit(x) = PSDM(x) – PSDM(x_org_), where x stands for the mutated sequence and x_org_ the original one, quantifying the PS ability difference between them. GA creates an initial population of random mutants, then iterates through selection, crossbreeding and mutation to recommend optimal single or few‐site amino acid mutations that match natural mutation patterns. The recommended mutants can be experimentally validated for PS ability, supporting the engineering of cell functions via regulated PS behavior (Figure S2) (Data was available in https://github.com/zly20020208/PScalpel.git).

### 2.2 PScalpel Exhibits Advanced Performance, Robustness, and Generalizability in Predicting the PS Behavior of Mutated Proteins

**FIGURE 2:**
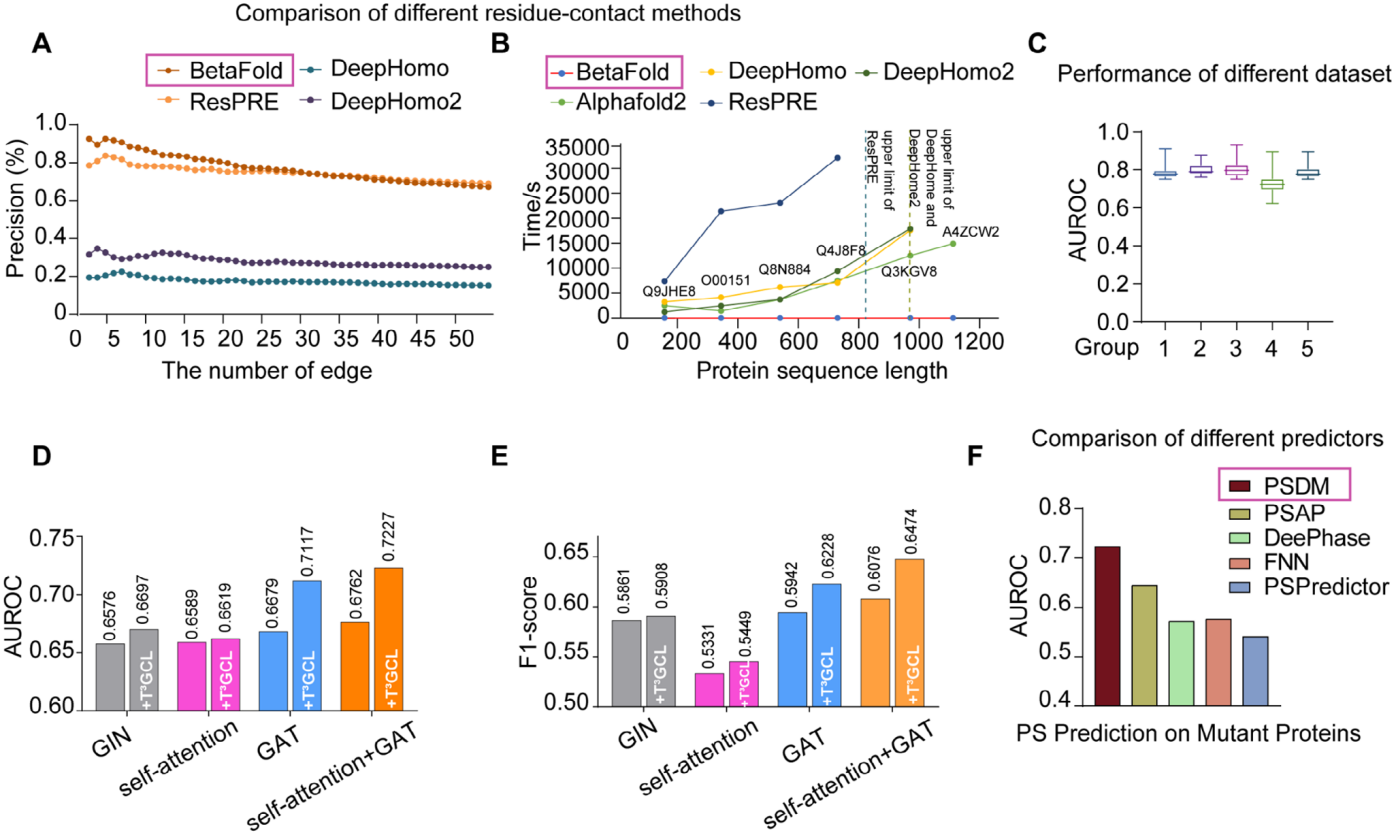
PScalpel exhibits advanced performance, robustness and generalizability in predicting the PS behavior of mutated proteins. (A) Comparison of 4 RRCP model including BetaFold, ResPRE, DeepHomo, and DeepHomo2 on the CASP14 dataset, which consisted of 34 realistic targets with experimental structures. The precision (%) was depicted as a function of the number of predicted contacts. (B) Efficiency comparison between BetaFold, AlphaFold2, ResPRE, DeepHomo, and DeepHomo2 for protein sequences of varying lengths. (C) The performance of BetaFold robustness by AUROC value, the 3D structural data of natural proteins obtained from experimental calculations were downloaded from the RCSB‐PDB database to construct the test dataset. (D,E) The T^3^GCL ablation study of PSDM was evaluated on PS dataset of mutated proteins. The performance of PSDM model was analyzed on AUROC value (D) and F1‐score (E) under each component. (F) The prediction performance of PSDM on mutated proteins analyzed by AUROC value compared with other 4 methods.

To validate the predictive performance of our model, we compared each module of PScalpel with the leading methods. Specifically, BetaFold was benchmarked against residue–residue contact prediction (RRCP) models including ResPre [39], DeepHome [40] and DeepHome2 [41], via top‐10 contact prediction on Critical Assessment of Structure Prediction 14 (CASP14) target T1024. CASP14 encompassed 84 experimental models across diverse structural groups (T1024‐T1101) [42]. Our results demonstrated that BetaFold maintained consistent top‐10 contact prediction accuracy, while control models showed distinct noncontact errors, confirming BetaFold's accuracy and robustness in residue–residue contact prediction (Figure S3A). Next, we evaluated the predictive performance of the corresponding proteins via 34 protein sequences from the CASP14 dataset. By scoring the performance of different models, area under the receiver operating characteristic (AUROC) values and F1 scores were obtained. As the inner module of the PScalpel, BetaFold performed well in terms of precision and efficiency prediction for proteins of varying lengths, which could provide informative structural descriptions (Figure 2A,B). In addition, the AUROC analysis clearly revealed the superior predictive ability of BetaFold (Figure S3B). Furthermore, we analyzed the actual 3D structure of the targeted proteins from the PDB by random grouping and obtained the AUROC and F1 score (Figure 2C, Figure S3C). These comparisons and evaluations demonstrated that BetaFold exhibited high accuracy, robustness, and superior predictive capabilities in predicting residue‒residue contacts and protein structures.

We next performed ablation studies to evaluate the efficacy of each component within the proposed PSDM module. Compared with other feature extraction techniques, T^3^GCL, as the core of PSDM, notably improved the feature extraction capability of the model, which operated comprehensively (Figure 2D,E). This also indicated that adding the protein structure predicted by the BetaFold improved the model's performance. Finally, we evaluated the protein PS property prediction performance in terms of the AUROC and F1 score for each of the five PS prediction methods, including PSDM, PSAP, DeePhase, the feed‐forward network‐based approach (FNN), and PSPredictor. All these methods performed well on natural proteins. However, for the mutated proteins, PSDM consistently outperformed other baselines with high significance, indicating that other tools were unsuitable for differentiating the PS propensity of mutated proteins (Figure 2F, Figure S3D).

### 2.3 PScalpel can Provide Reliable PS Ability Engineering Strategy

**FIGURE 3:**
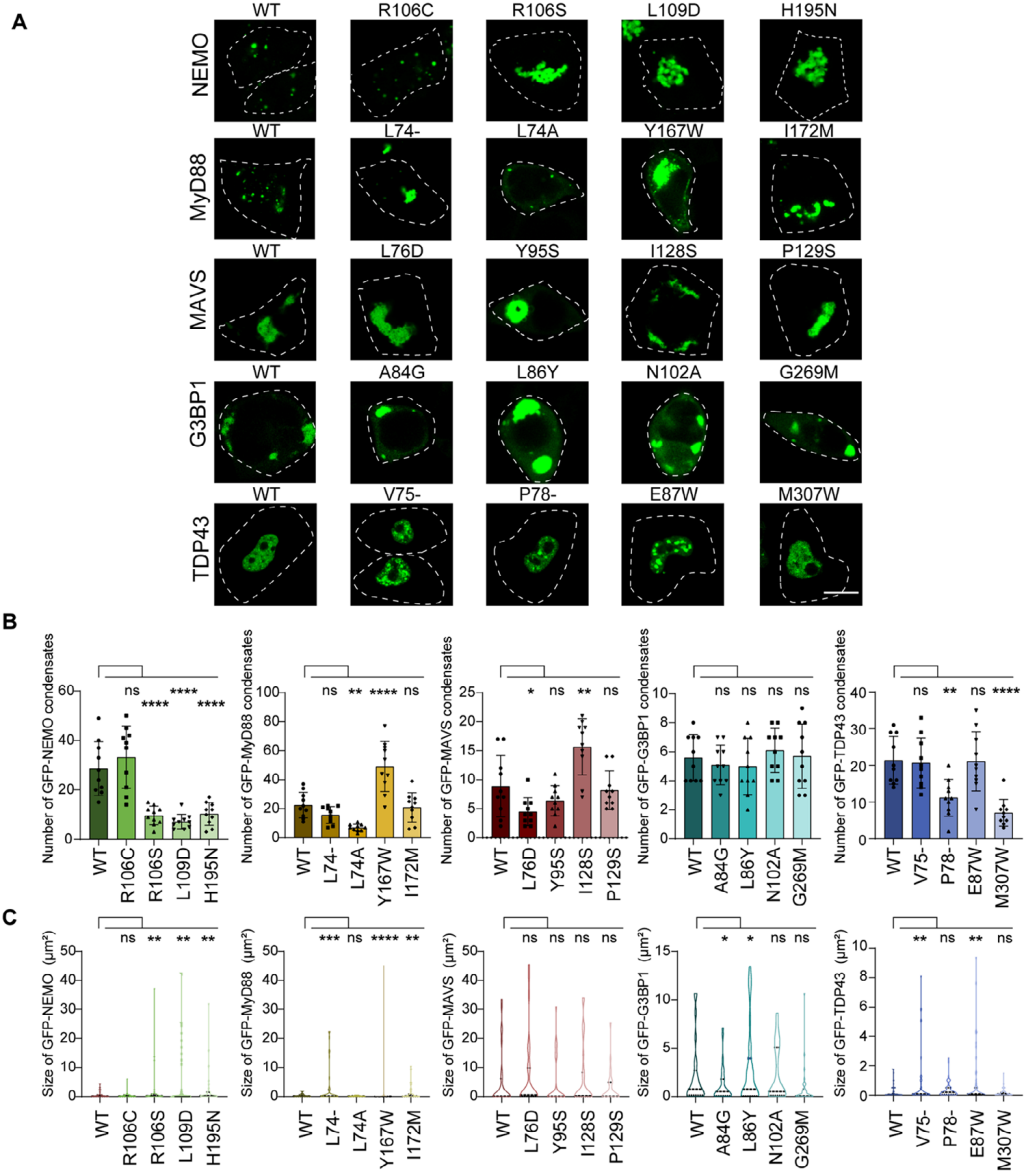
PScalpel provides reliable PS ability engineering strategy. (A) Fluorescence microscopy images showed wild type (WT) NEMO, MyD88, MAVS, G3BP1, TDP43, and their mutants as labeled (e.g., R106C means Arginine at position 106 mutated to Cysteine). The proteins were overexpressed in HEK‐293T cell line with plasmids. The cell boundaries were marked with white dashed lines. Scale bar, 10 µm. (B,C) The number of WT GFP‐tagged indicated proteins and their mutants condensates were counted from n = 10 views (B), and the size of condensates was measured from n = 100 condensates (C). Both analyzes were under the condition of proteins overexpressed in HEK‐293T cell line with plasmids. Data in (B) were expressed as mean ± SD of indicated samples for each condition. Data in (C) were expressed as median and quartiles of indicated samples for each condition. *p < 0.05, **p < 0.01, ***p < 0.001, ****p < 0.0001, ns, not significant (one‐way ANOVA). Similar results were obtained for three independent biological experiments in (A).

The PScalpel was capable of engineering any protein to enhance its PS ability. To verify the model's universality and reliability, we selected 8 proteins that play important roles in innate immunity and possess PS capacity as verification candidates, including NEMO, MAVS, G3BP1, MyD88, TDP43, etc. [43, 44, 45, 46, 47, 48]. We chose the mutation strategies with the highest prediction score and verified them through biological experiments. From the experimental results, it could be seen that the single site mutation provided by the PScalpel was able to change the protein's PS ability (Figure 3A, Figure S4A). By comparing the PS abilities of indicated wild type (WT) protein and its mutants based on the number and volume of intracellular condensates, we found that most of the mutagenesis strategies yielded favorable outcomes (Figure 3B,C, Figure S4B,C). We statistically analyzed the results of altered PS ability from these protein experiments, quantified the positive and false‐positive outcomes, and found that prediction accuracy for different proteins varied between 50%–100%, and the average false‐positive rate was less than 25% (Figure S4D,E).

**FIGURE 4:**
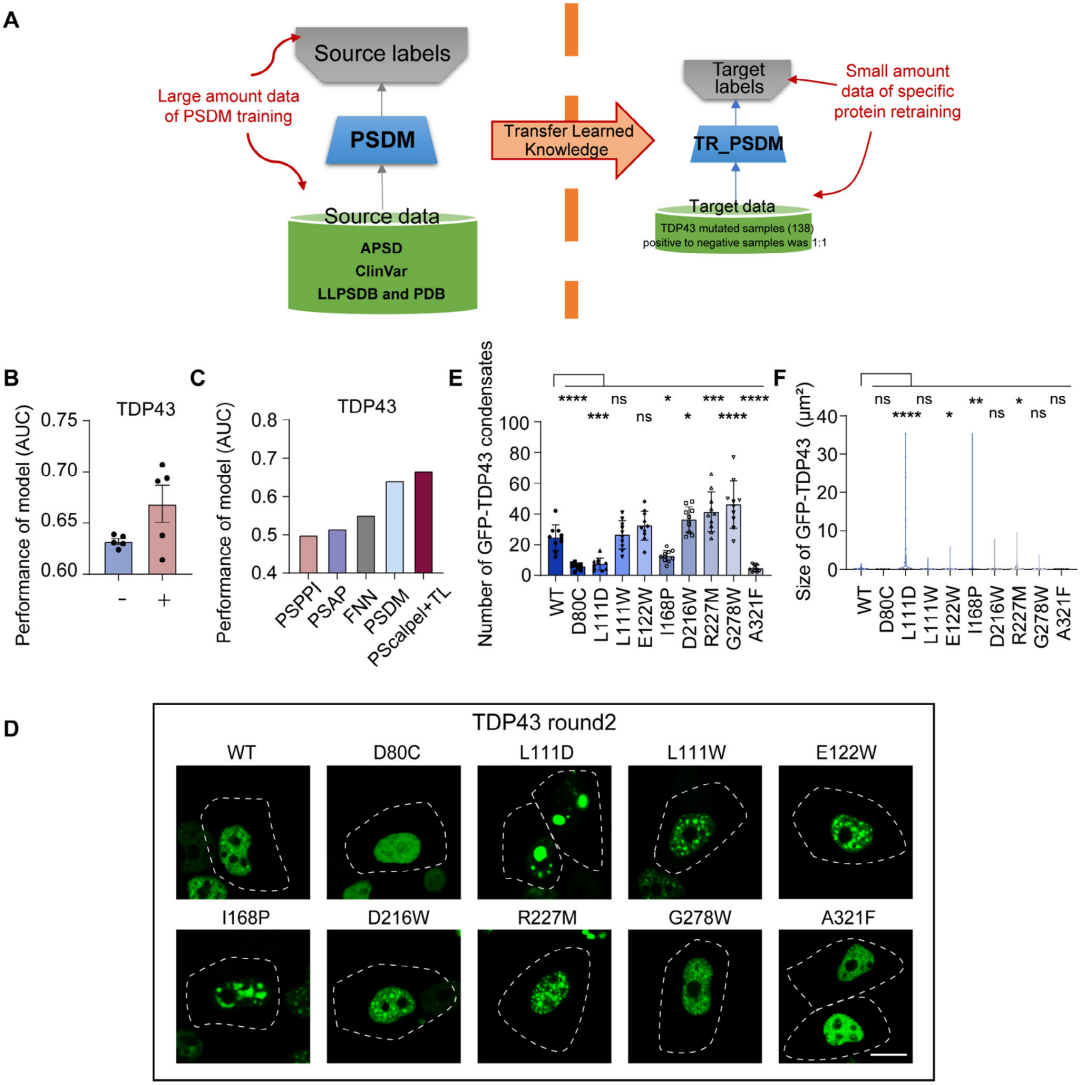
TL method enhances model performance on specific protein. (A) The schematic of TL method used on TDP43 retraining. (B) Performance comparison of PScalpel on TDP43 before and after retraining. (C) Performance comparison of PScalpel with other methods on the domain of TDP43. (D) Fluorescence microscopy images showed wild‐type (WT) TDP43 and its mutants as labeled (such as V75‐ means Valine (V) at position 75 deletion) in round 2. Round 2 was predicted by TR_PSDM, retraining with TL method. TDP43 was overexpressed in HEK‐293T with plasmids. Scale bar, 10 µm. (E‐F) The number of WT GFP‐TDP43 and mutations condensates was counted from n = 10 views (E), and the size of GFP‐TDP43 condensates was measured from n = 100 condensates (F). Both analyses were under the condition of proteins overexpressed in HEK‐293T cell line with plasmids. Data in (B) were expressed as mean values ± SEM were expressed of n = 5 independent experiments. Data in (E) were expressed as mean ± SD of indicated samples for each condition. Data in (F) were expressed as median and quartiles of indicated samples for each condition. *p < 0.05, **p < 0.01, ***p < 0.001, ****p < 0.0001, ns, not significant (one‐way ANOVA). Similar results were obtained for three independent biological experiments in (D).

However, the experimental validation accuracy for some protiens like TDP43 prediction still fell short of expectations. To address this issue and further enhance the model's predictive performance for specific proteins, we introduced a Transfer Learning (TL) method. TL mitigated overfitting in small sample scenarios by reusing deep feature representations pre‐trained on large‐scale source domain data [49, 50]. The low‐level features learned by the PSDM model in general tasks (PS prediction for mutant proteins) exhibited strong transferability, providing a favorable initialization in the high‐dimensional semantic space for TDP43 application (PS prediction for specific proteins) (Figure 4A). This method reduced the model's dependency on limited target data. Meanwhile, freezing partial underlying parameters or adopting fine‐tuning strategies during the transfer process preserved general feature extraction capabilities, thereby improving the model's generalization performance under small‐sample conditions. Here, the data used for TL model retraining were the site mutations of TDP43, such as A326P (means alanine at position 326 mutated to proline) mutation, which has been reported in previous studies to alter the PS ability of TDP43 [11]. During the retraining process, a total of 138 mutated samples were used, and the ratio of positive to negative samples was 1:1 (Data S2). Specifically, whether the mutation of the TDP43 led to a significant change in its PS ability was used as the basis for determining positive and negative samples, respectively. After the existing mutation sites of TDP43 were input into the TL model, the performance of PScalpel for TDP43 was significantly promoted (Figure 4B). Compared with other methods for the TDP43 task, TL model training enabled PScalpel to achieve better performance (Figure 4C). In addition, a significant improvement in accuracy was observed in the biological experiment results of the TDP43 before and after retraining. Among the 9 mutants in the round 2, TDP43 mutants of L111D, L111W, E122W, I168P, D216W, R227M, and G278W showed significant enhancements in their PS abilities (Figure 4D–F). In conclusion, these results indicated that retraining the model using existing mutation sites with known effects could improve prediction accuracy for specific proteins. This enhancement established PScalpel as an effective tool for predicting protein PS ability and guiding site‐specific modification, with advanced performance, robustness, and generalizability.

### 2.4 PScalpel Provides a Precise Engineering Strategy for Regulating the PS Ability of the Immune Protein cGAS

cGAS, the most critical DNA sensor in innate immunity, undergoes PS when it senses and interacts with DNA, thereby regulating downstream signal transduction [30]. Therefore, modulating its PS ability enables precise intervention in the cGAS‐STING signaling pathway. Single amino acid engineering of cGAS using the PScalpel avoided extensive trial‐and‐error, laying the foundation for applying this precision engineering approach to immune cell reprogramming.

**FIGURE 5:**
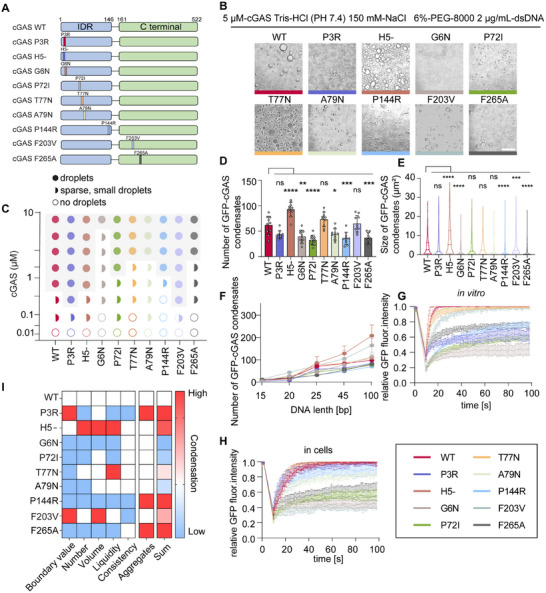
PScalpel provides a precise engineering strategy for regulating cGAS PS ability. (A) Schematic diagram of full‐length human cGAS marked with indicated single amino acid mutations (e.g., P3R means Proline at position 3 mutated to Arginine; H5‐ means Histone at position 5 is deleted). (B) Bright field microscopy images of 5 µM wild type (WT) GFP‐cGAS and its mutants show differences in droplet formation. Scale bar, 10 µm. (C) Phase diagram showing changes in the boundary value of WT cGAS and its mutants. Protein concentrations were used as indicated, incubated with 45 bp dsDNA (2 µg mL^−1^) in PS buffer for 15 min at 37°C. Sparse, small droplets mean the condensate can almost impossible to maintain a certain scale like WT GFP‐cGAS at 0.1 µM in Figure S5B. (D,E) The number of WT GFP‐cGAS and mutations condensates was counted from n = 10 views (D) and the size of GFP‐cGAS condensates was measured from n = 200 condensates (E). Both analyzes were under the condition of cGAS (5 µM) mixture with 45 bp dsDNA (2 µg mL^−1^) in PS buffer for 15 min at 37°C. (F) The number of WT GFP‐cGAS and mutations condensates in PS buffer containing different length of dsDNA (2 µg mL^−1^) under the condition of cGAS (5 µM) for 15 min at 37°C. (G) Representative images of confocal microscopy showing WT GFP‐cGAS and mutations recombinant proteins (5 µM) mixed with 45 bp dsDNA (2 µg mL^−1^) and incubated in PS buffer at 37°C. Bleaching was performed at the indicated time points and the recovery occurred at 37°C. Fluorescence intensity analysis of FRAP from n = 6 condensates over 100 s time course. Scale bar, 10 µm. (H) HEK‐293T cells expressing WT GFP‐cGAS and mutants and transfected with HT‐DNA (2 µM) for 12 h were placed on the dishes at 37°C. After seeding, bleaching of the cGAS foci was performed and quantification of FRAP of GFP‐cGAS condensate was analyzed. The start of recovery after photobleaching was defined as 0 s. Representative images of n = 6 cells were shown. Scale bar, 10 µm. (I) Scoring diagram for the PS ability of cGAS mutants. Sum means Summary. Data in (G, H) were expressed as mean values ± SEM were expressed of n = 6 independent experiments. Data in (D, F) were expressed as mean ± SD of indicated samples for each condition. Data in (E) were expressed as median and quartiles of indicated samples for each condition. *p < 0.05, **p < 0.01, ***p < 0.001, ****p < 0.0001, ns, not significant (one‐way ANOVA). Similar results were obtained for three independent biological experiments in (B,C).

Here, a total of 9 high‐scoring mutants of the cGAS were predicted and validated, most of which are located in the IDR region of cGAS (Figure 5A). To assess the PS ability of cGAS, we generated recombinant proteins of GFP‐cGAS with mutations by *E.coli* expression system and equalized their concentrations (Figure S5A). After mixing the cGAS mutants with dsDNA, micrometer‐sized condensates formed within 2 min and stabilized after 15 min, obvious differences in the condensate size were observed by microscopy, indicating that these mutants differentially impacted the PS ability of cGAS (Figure 5B). These results validated the feasibility of single site amino acid engineering for cGAS.

To further define the effects of the mutation on the PS capability of cGAS, we established a comprehensive evaluation system both in vitro and in cells. First, P3R and F203V mutations decreased the phase boundary values, indicating a lower protein concentrations for PS. In the contrast, G6N mutant showed a markedly higher phase boundary value, meaning it required a higher protein concentration for PS process (Figure 5C, Figure S5B). Moreover, we noticed that H5 deletion (H5‐) and F203V mutation enhanced the condensate size and poliferation, and P3R, P144R, and F265A formed irregular aggregates with reduced size and number (Figure 5D,E, Figure S5C). As DNA valency is the driving force of cGAS‐DNA phase separation, longer DNA with higher valency tends to promote more condensate formation without sequence specificity [51, 52]. We incubated cGAS and its mutants with different lengths of DNA in PS buffer (Figure S5D). H5‐ and F203V mutants showed enhanced PS ability specifically with longer dsDNA, while the G6N and F265A mutants exhibited reduced PS ability under all conditions, forming fewer and smaller condensates (Figure 5F). In vitro Fluorescence Recovery After Photobleaching (FRAP) assays revealed that P3R, G6N, P72I, P144R, and F265A mutants exhibited reduced dynamic properties (Figure 5G, Figure S6A). The low mobility of P3R, P144R, and F265A was likely attributed to aggregate formation driven by enhanced PS ability. Furthermore, cellular FRAP assays revealed that cGAS mutants P3R, T77N, P144R, and F203V exhibited dynamic properties opposite to those observed in vitro (Figure 5H, Figure S6B). By comparing the PS capacity of WT cGAS, these mutants could be classified into two categories: those with significantly altered PS ability, and those forming aggregates due to excessive PS capacity. Specifically, H5‐, G6N, P72I, T77N, A79N, and F203V showed significant PS changes, and P3R, P144R, F265A formed aggregates owing to hyperactive PS capacity (Figure 5I, Figure S7).

Collectively, the validation results of cGAS mutants demonstrated the utility of PScalpel in regulating protein PS ability, laying a foundation for subsequent investigations into cGAS mutant immune functions and cellular engineering.

### 2.5 PS‐Associated Single Amino Acid Mutations in cGAS Changed its Function in Cells

**FIGURE 6:**
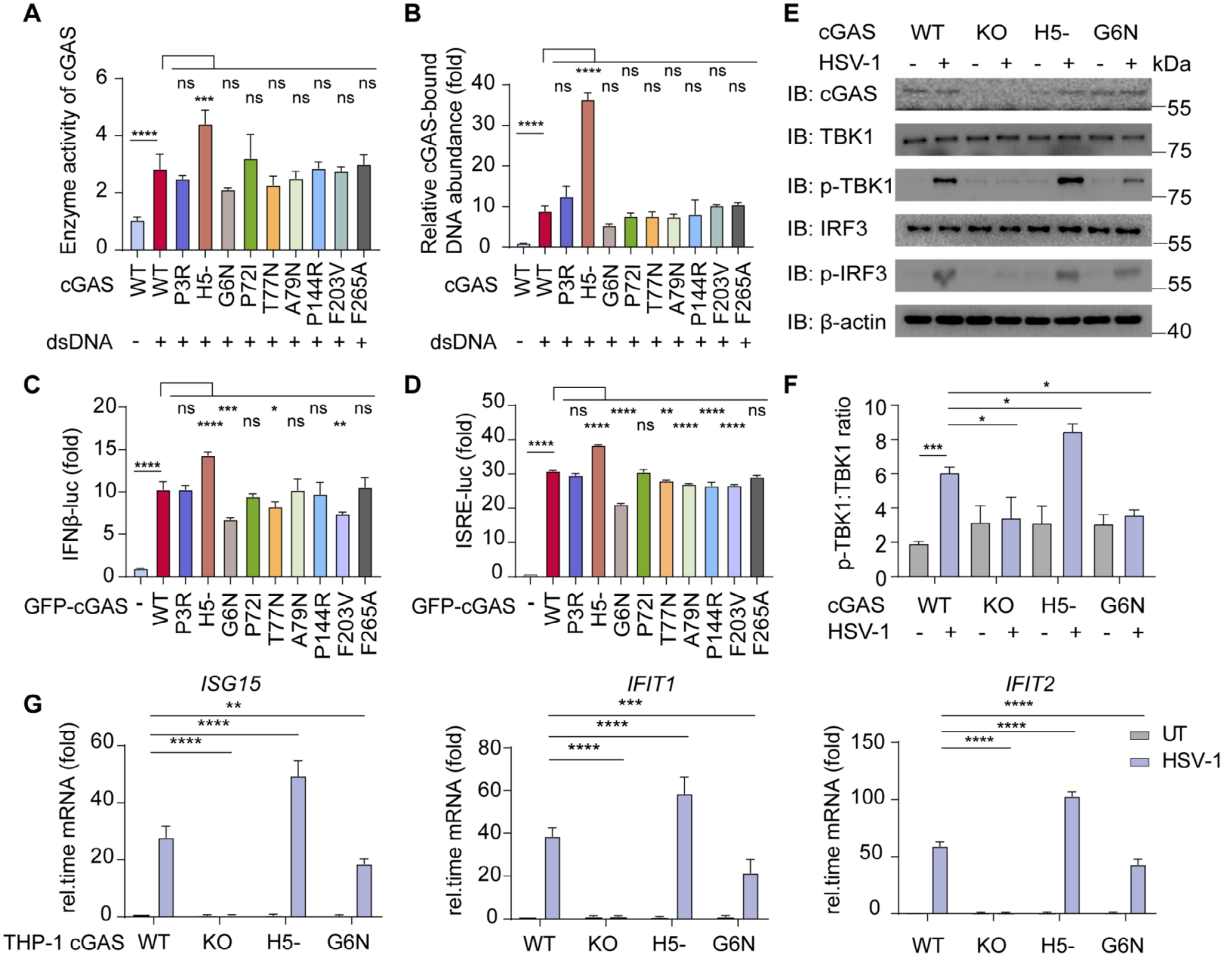
cGAS H5‐ and G6N mutations affect their functions in type I IFN signaling. (A) In vitro cGAMP processing ability of recombinant wild type (WT) cGAS and its mutants (10 µM) incubated with 45 bp dsDNA (2 µM), along with ATP and GTP. (B) HEK‐293T cells expressed with WT cGAS and its mutants were transfected with mCherry plasmid (2 µg mL^−1^) as plasmid DNA for 2 h before harvest. Cell lysates were collected, immunoprecipitated with A+G beads together with cGAS antibody, followed by qRT‐PCR analysis of extracted DNA to detect cGAS‐bound plasmid DNA (*mCherry*) abundance. (C) HEK‐293T cells transfected with IFNβ luciferase reporter (IFNβ‐luc) and TK‐luc, WT GFP‐cGAS and its mutants to HEK‐293T‐STING cell line, treated with HT‐DNA (2 µM) for 12 h. Cell lysates were collected and luciferase assay was performed. (D) HEK‐293T cells transfected with ISRE luciferase reporter (ISRE‐luc) and TK‐luc, WT GFP‐cGAS and its mutants to HEK‐293T‐STING cell line, treated with HT‐DNA (2 µM) for 12 h. Cell lysates were collected and luciferase assay was performed. (E) Immunoblot analysis of WT, *cGAS* KO, and indicted cGAS mutants expressing THP‐1‐derived macrophages treated with HSV‐1 (MOI = 1) for 24 h. (F) Quantification of the expression levels of p‐TBK1 shown in (E). (G) qRT‐PCR with reverse transcription analysis of *ISG15, IFIT1* and *IFIT2* mRNA level of WT, *cGAS* KO, and indicted cGAS mutant expressing THP‐1‐derived macrophages treated with HSV‐1 (MOI = 1) for 24 h. Data in (A‐D, F‐G) were expressed as mean values ± SD were expressed of n = 3 independent biological experiments. *p < 0.05, **p < 0.01, ***p < 0.001, ****p < 0.0001, ns, not significant (one‐way ANOVA). Similar results were obtained for three independent biological experiments in (E).

To characterize the molecular function of cGAS mutants with altered PS ability, we performed cellular assays to evaluate cGAS enzymatic activity, DNA binding affinity and downstream signaling activation. PS ability of cGAS plays a vital role in cGAS‐dependent DNA recognition and signaling, modulating the PS ability of cGAS could affect cGAS‐mediated type I interferon (IFN) signaling [30]. First, we detected the enzyme activity of cGAS with different mutants by checking ATP and GTP consumption and found that the cGAS H5‐ mutant presented increased ATP/GTP consumption in vitro, indicating the enhancement of enzymatic activity (Figure 6A). We next performed a cGAS‐bound DNA experiment and found that the H5‐ mutant presented increased DNA binding ability (Figure 6B). To determine whether cGAS mutations regulate immune responses, we treated STING‐expressing HEK‐293T cells with cGAS mutants followed by HT‐DNA stimulation. IFN and ISRE luciferase reporter assays revealed that the H5‐ mutant potently enhanced type I IFN signaling activation (Figure 6C,D). We also noticed the G6N mutant reduced type I IFN signaling activation by IFN and ISRE luciferase reporter assays (Figure 6C,D).

To further test whether the cGAS H5‐ and G6N mutants have stronger and weaker immune functions, we detected the cGAS‐mediated type I IFN signaling pathway in STING‐expressing HEK‐293T cells transfected with plasmids encoding cGAS mutants of H5‐ and G6N, along with DNA virus Herpes Simplex Virus‐1 (HSV‐1) treatment. TBK1 and IRF3 are the key components of the cGAS‐STING signaling pathway, and their phosphorylation levels reflect pathway activation [53]. Immunoblotting revealed that the cGAS H5‐ mutation promoted the HSV‐1 induced phosphorylation level of TBK1 and IRF3, whereas the G6N mutation decreased the phosphorylation level of TBK1 and IRF3 (Figure S8A). The mRNA levels of interferon‐stimulated genes (ISGs) induced by HSV‐1 were also increased in cGAS H5‐ mutant‐expressing cells, whereas the expression of those ISGs was inhibited in G6N mutant‐expressing cells (Figure S8B). To further elucidate the functions of H5‐ and G6N mutations, we generated a *cGAS* knockout (KO) THP‐1 cell line (a human monocyte/macrophage cell line) and reintroduced H5‐ and G6N mutations to generate *cGAS^H5−^* and *cGAS^G6N^* THP‐1 cells, respectively (Figure S8C). Next, we derived THP‐1 cells to macrophages to investigate whether H5‐ and G6N mutations in cGAS affect the cGAS‐induced antiviral response after stimulation of *cGAS*‐KO cells with HSV‐1. Compared with WT THP‐1‐derived macrophages, *cGAS^H5−^* THP‐1‐derived macrophages were more sensitive to virus infection, as indicated by increased phosphorylation levels of TBK1 and IRF3, whereas *cGAS^G6N^* THP‐1‐derived macrophages were insensitive to virus infection and presented reduced phosphorylation levels of TBK1 and IRF3 (Figure 6E, F and Figure S8D). Similarly, increased mRNA levels of ISGs in *cGAS^H5−^* THP‐1‐derived macrophages and restricted expression levels of ISGs in *cGAS^G6N^* THP‐1‐derived macrophages were observed, which were consistent with previous results (Figure 6G). Taken together, these findings demonstrated that the cGAS H5‐ and G6N mutants changed the immune function of cGAS through alterations in the PS properties.

### 2.6 Fine‐Tuning of Immune Responses Through cGAS PS Mutations

**FIGURE 7:**
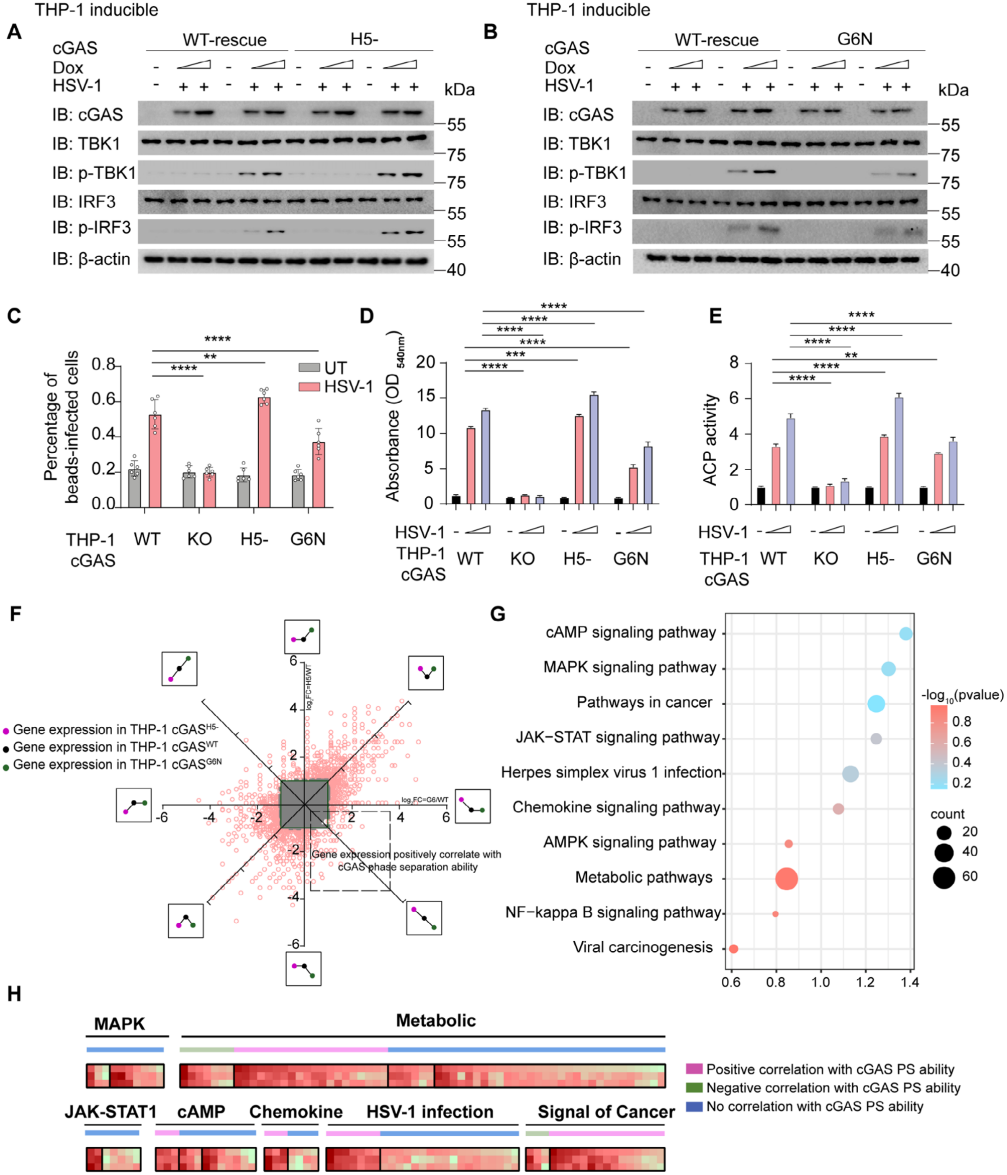
cGAS PS mutations impact the immune functions of macrophages and precisely regulate distinct downstream responses. (A) Immunoblot analysis of *cGAS ^wild type(WT)/H5−^* inducible THP‐1‐derived macrophages treated with doxycycline (Dox, 200 and 400 ng mL^−1^) for 24 h, then infected with HSV‐1 (MOI = 1) for 24 h. (B) Immunoblot analysis of *cGAS ^WT/G6N^* inducible THP‐1‐derived macrophages treated with doxycycline (Dox, 200 and 400 ng mL^−1^) for 24 h, then infected with HSV‐1 (MOI = 1) for 24 h. (C) Phagocytosis of WT, *cGAS* KO, and indicted cGAS mutant expressing THP‐1‐derived macrophages labeled with carboxyfluorescein diacetate, fluorescent microsphere beads incubated macrophages for 30 min, the percentage of beads‐infected cells was counted and analyzed from n = 30 cells. (D) Pinocytosis of WT, *cGAS* KO, and indicated cGAS mutant expressing THP‐1‐derived macrophages labelled with Neutral red dye, the activity was detected by absorbance in 540 nm. (E) Acid phosphatase (ACP) activity of WT, *cGAS* KO, and indicted cGAS mutant expressing THP‐1‐derived macrophages through p‐Nitrophenyl Phosphate (pNPP) measurement, detected by absorbance at 405 nm. (F) Distribution of different expression tendencies of cGAS PS ability related genes between cGAS *^WT/H5−^ ^/G6N^* expressed THP‐1 via HSV‐1 (MOI = 1) infection for 24 h. The x axis represents the log_2_FC value of cGAS*^G6N^* versus cGAS*^WT^*, while the y axis represents the log_2_FC value of cGAS*^H5−^* versus cGAS*^WT^*. The three colored annotations on the left represented the change trend of gene expression in THP‐1 cGAS*^H5−^*, cGAS*^WT^*, cGAS*^G6N^* respectively. The eight surrounding boxes showed the trend of gene change in the THP‐1 cells of the above genotypes. (G) KEGG pathway analysis of cGAS PS‐related genes from (E), the size of bubbles was measured by counts and colored with ‐log_10_(*p* value) degree. (H) With grouped different genes expression tendencies in Figure 7F into positive, negative and no correlation with cGAS PS genes groups. Transcriptome sequencing analysis of these genes labeled by pathway information. Data in (C) were expressed as mean ± SD of indicated samples for each condition. Data in (D,E) were expressed as mean values ± SD were expressed of n = 3 independent biological experiments. **p < 0.01, ***p < 0.001, ****p < 0.0001 (one‐way ANOVA). Transcriptome sequencing data are mean ± standard deviation of three independent experiments. Similar results were obtained for three independent biological experiments in (A,B).

Next, we sought to generate engineered macrophage whose functions were regulated by PS ability upon cGAS mutants. cGAS‐mutant‐inducible THP‐1 cell lines were constructed from *cGAS‐*KO THP‐1 cells. Our results demonstrated that *cGAS^H5−^*‐inducible THP‐1‐derived macrophages presented increased phosphorylation levels of TBK1 and IRF3, indicating enhanced activation of type I IFN signaling (Figure 7A). Furthermore, the mRNA levels of ISGs, including *ISG15*, *IFIT1* and *IFIT2*, were significantly greater in *cGAS^H5−^* inducible THP‐1‐derived macrophages than in *cGAS^WT^* THP‐1‐derived macrophages (Figure S9A). Conversely, *cGAS^G6N^* inducible THP‐1‐derived macrophages presented reduced activation of type I IFN signaling with lower phosphorylation levels of TBK1 and IRF3 (Figure 7B). The mRNA levels of several ISGs were also inhibited in *cGAS^G6N^*‐inducible THP‐1‐derived macrophages (Figure S9B). These results demonstrated that our PS alteration mutants successfully regulated cGAS immune and antiviral functions, allowing fine‐tuning of downstream immune responses via single‐site mutations.

Additionally, we examined the physiological function of THP‐1‐derived macrophages to determine the changes regulated by cGAS PS ability. Basically, *cGAS^WT^* and *cGAS^mutant^* THP‐1‐derived macrophages had similar cell viability under normal conditions (Figure S9C, D). Compared with *cGAS^WT^* THP‐1‐derived macrophages, *cGAS^H5−^* THP‐1‐derived macrophages exhibited enhanced phagocytic ability, as evidenced by the capture of a greater number of fluorescent beads. Conversely, *cGAS^G6N^* THP‐1‐derived macrophages demonstrated weaker phagocytic ability (Figure 7C, Figure S9E). Neutral red staining also revealed enhanced pinocytosis activity in *cGAS^H5−^* and decreased pinocytosis activity in *cGAS^G6N^* THP‐1‐derived macrophages (Figure 7D). pNPP analysis revealed that *cGAS^H5−^* THP‐1‐derived macrophages presented increased lysosomal enzyme activity, whereas *cGAS^G6N^* THP‐1‐derived macrophages presented reduced lysosomal enzyme activity (Figure 7E). These findings revealed that the alteration of the PS ability of cGAS mutations affected not only type I IFN signaling but also the physiological functions of macrophages.

To further characterize the comprehensive map of functional changes regulated by cGAS PS ability, we performed transcriptome sequencing of *cGAS^WT^, cGAS^H5−^* and *cGAS^G6N^*‐expressing THP‐1‐derived macrophages. Through comparative analysis, we identified 1059 genes with significant differences [log_2_(FC)>1] among the three cell lines (Figure 7F). Intriguingly, most of the differently expressed genes in *cGAS^H5−^* and *cGAS^G6N^* cells exhibited opposite transcriptional patterns compared with those in *cGAS^WT^* cells (in the first and third quadrants). These results highlighted the crucial roles of the cCAS PS capacity in maintaining cellular processes and homeostasis. Gene Ontology (GO) enrichment analysis of genes correlated with cGAS's PS ability revealed significant differences in genes related to cholesterol homeostasis, inflammatory response, and immune response in the biological process category (Figure S9F). Functional enrichment analysis with p values suggested that cGAS PS alteration modulated ion binding, ATP binding and DNA binding (Figure S9G). Kyoto Encyclopedia of Genes and Genomes (KEGG) analysis of genes correlated with cGAS's PS ability revealed that the genes whose expression significantly differed were enriched in metabolic, cAMP and viral infection pathways (Figure 7G). To further systematically confirm whether the PS ability of cGAS could manipulate downstream immune responses, we analyzed the tendencies of genes whose expression was positively or negatively correlated with cGAS function. Notably, the immune function of cGAS regulated by PS alteration was not only correlated with viral infection but also associated with inflammation and cellular homeostasis (Figure 7H). Overall, our data provided evidence that the single amino acid editing strategy of cGAS suggested by PScalpel could affect its PS properties, subsequently altering the immune function of cGAS to determine distinct downstream immune responses. Furthermore, we manipulated the PS ability of cGAS via relatively consistent protein expression to achieve precise regulation of the targeted signaling network, which inspired us to investigate the significance of PScalpel in medical biomolecular engineering.

## 3 Discussion

In recent decades, the important role of phase separation has gradually been revealed, and it profoundly affects biological processes such as metabolism, development and immunity [2]. The prediction and engineering of protein PS properties can accelerate mechanistic research, biological tool development, and disease analysis. For example, the use of engineering condensates to study phosphorylation specificity [54], enhancing CRISPR transcriptional activity with PS components [55], and confirming disease‐associated PS proteins [56]. Although there is a massive demand for engineering protein PS properties to study and regulate cellular processes, manipulating PS ability through single amino acid alterations remains a challenge. In this study, we developed PScalpel, the first computational tool designed to propose tailored engineering strategies for protein PS ability, thereby expanding avenues for biological process regulation and offering innovative approaches for biosynthetic applications. For biologists, especially those researchers who aimed to conduct in‐depth studies and apply the protein PS function, the PScalpel model presented here serves as an optimal resource. It empowers users to alter the PS ability of target proteins to induce functional changes independent of enzymatic activity, strictly following user‐defined specifications.

PScalpel is a machine learning framework that integrates graph contrastive learning with protein structural data to guide the alteration of PS properties through single amino acid engineering. PScalpel comprises 2 advanced approaches, including BetaFold, T^3^GCL. The BetaFold model is an efficient RRCP algorithm that accurately extracts protein graph structure features, addressing challenges in identifying mutations within similar protein variants. The T^3^GCL method is a novel approach that extends GCN applications, overcoming data limitations in biological research. BetaFold and T^3^GCL jointly constitute the PSDM model, which is capable of distinguishing proteins' PS ability, and together with GA, they play a role in recommending alteration strategies. We compared the PScalpel with other models and validated its universality in eight immune‐related proteins. These results underscored the model's excellent performance, and further statistical analysis of altered PS ability data from these experiments quantified true and false positives, revealing the prediction accuracy ranging from 50% to 100% across proteins with an average false‐positive rate below 25%.

Amino acids, as the basis of proteins, are crucial for their function, and slight changes may lead to functional alterations and diseases such as sickle cell anemia [57, 58]. Single‐site mutations affecting PS ability are known to influence condensate properties, resulting in degenerative diseases [11, 59]. However, the number of modification strategies based on a single amino acid increases exponentially with increasing protein length and candidate amino acid type. Current tools struggle to predict how single amino acid mutations impact protein PS propensity, as they are primarily designed to analyze natural proteins with substantially divergent sequences rather than mutant variants. In this study, PSDM was constructed to discern the subtler changes of mutant proteins combined the graph structural features. Additionally, we introduced TL method to improve accuracy for specific proteins, such as TDP43, through which we validated its effectiveness. The TL method adapts deep learning models pre‐trained on large‐scale general datasets to various downstream tasks by fine‐tuning with limited task‐specific data [60]. The method has demonstrated significant utility in diverse biological contexts, such as gene regulatory network and drug target discovery [61], as well as improving medical image recognition accuracy [62]. This approach reduces the dependency on large annotated datasets for target tasks, cutting training costs and time, while enhancing model generalization and learning efficiency through knowledge transfer. It is particularly suitable for protein PS mutation scenarios, where specific proteins data are limited.

Engineered immune cells refer to the modification of immune cells to enable them to recognize and respond to disease states, ultimately achieving therapeutic purposes [63]. Over the past few decades, synthetic biology has rationally constructed signaling components to generate effective cell therapies such as CAR‐T, which successfully entered clinical practice [64]. However, balancing efficiency and safety in therapeutic strategies still remained a challenge. Phase separation drove functionally associated proteins to form membraneless compartments in cells, conditionally controlled substance influx and efflux, and created a rapid‐response hub. In this hub, any minor alteration was amplified and exerted far‐reaching effects on the entire system. Based on this feature, methods to control cellular vital activities through phase separation have gradually come into view of researchers. For example, compartmentalized enzymatic reaction centers were constructed in bacteria to improve biosynthetic yield [65], and modification of CAR element to enhance functional stability [66], both cases leveraging PS‐mediated modularization by adding PS components. Here, we proposed to precisely engineer immune cells by single amino acid modification of the PS function of key immune components. Given the relatively independent reaction environment inherent in PS, we believed this approach can offer better manipulability. cGAS, the paramount DNA recognition receptor in innate immunity, triggers the cGAS‐STING signaling cascade to orchestrate multifaceted immune responses against viral infections and tumor progression, with its PS ability essential for efficient DNA sensing and functional homeostasis [67]. In this work, the PScalpel was employed to identify 9 mutants of cGAS with significantly altered PS ability, 2 of which were employed to construct engineered immune cells for cGAS‐based cellular manipulation via PS modulation. We analyzed the physiological functional data from THP‐1 cGAS rescued cell lines, and ensured that the protein level of cGAS and its mutants across three cell groups were equal, thus confirming that those mutations only altered cGAS's PS ability. Consistent with previous studies, manipulation of cGAS function directly drives downstream transcriptional changes [68, 69], which further elucidates the critical role of cGAS PS ability in the regulation of immune homeostasis in macrophages.

In summary, we designed a novel PS modification guide, PScalpel, and validated its accuracy and practicality through biological experiments. Although the model had not fully established the link between protein function and PS ability, the ongoing advancements in related research would reveal more effects of single amino acid mutations on protein functions, which may help overcome these limitations in the future. Additionally, owing to the universality of the PScalpel, this model could also be used to guide the non‐immune cells engineering, meeting the diverse experimental needs of researchers. We believe that PScalpel can accelerate the application of PS modification and achieve precise regulation of targeted molecules and cells.

## 4 Experimental Section

### 4.1 BetaFold

Improvements to the Transformer architecture have been made to increase its suitability for extracting potential associations between amino acids in protein sequences. The detailed methods are provided in the Supporting Information.

### 4.2 T^3^GCL

T^3^GCL was developed by closely associating with target data and jointly learning consistent information in the contrastive learning field. The detailed methods are provided in the Supporting Information.

### 4.3 PSDM

The PSDM is a novel module developed to screen the optimal mutants for PS alteration demand. The detailed methods are provided in the Supporting Information.

### 4.4 Cell Culture

HEK‐293T (RRID:CVCL_0063) and Vero E6 (RRID:CVCL_0574) cells obtained from the Cell Bank of the Chinese Academy of Sciences (Shanghai, China) were cultured in DMEM (Corning) supplemented with 10% (vol/vol) foetal bovine serum (Gibco) and 1% L‐glutamine (Gibco) and incubated in a 37°C chamber with 5% CO_2_ (Thermo Fisher Scientific). THP‐1 (RRID:CVCL_0006) cells obtained from the Cell Bank of the Chinese Academy of Sciences (Shanghai, China) were cultured in RPMI 1640 (Gibco) supplemented with 10% (vol/vol) fetal bovine serum (FBS), 1% penicillin‒streptomycin (Gibco, 1:100) and 1% L‐glutamine (Gibco) and incubated in a 37°C chamber with 5% CO_2_ (Thermo Fisher Scientific). Before stimulation, the THP‐1 cells were differentiated into macrophages (THP‐1‐derived macrophages) via treatment with 100 nM phorbol‐12‐myristate‐13‐acetate (PMA) (P8139, Sigma) for 16 h. After PMA treatment, the macrophages were allowed to rest for 48 h before stimulation. Mycoplasma contamination has been tested negative.

### 4.5 Generation of KO Cell Lines

After the THP‐1 cells were seeded, the medium was replaced with DMEM containing polybrene (8 µg mL^−1^) (Sigma‒Aldrich) lentiviral vector encoding Cas9 and small guide RNA (sgRNA) for 48 h. The cells were selected with puromycin (Sigma‒Aldrich). The sequences of the sgRNAs targeting the indicated genes were obtained from Sangon (Shanghai, China) and are shown below:

**Table:** 

Targets	Sequence (5'‐3')
*GFP*	CATGCCGAGAGTGATCCCGG
*cGAS* sgRNA #1	CACCCACGCAGTTATCAAAGCAG
*cGAS* sgRNA #2	CACCCGGCCCCCATTCTCGTACGG

### 4.6 Generation of Stable Expression Cell Lines

For the stable expression of STING, cGAS and its mutants, lentiviral particles were produced by transfecting HEK‐293T cells with the STING‐lentiviral transfer vector together with the pLP1, pLP2, and Plp/VSVG vectors. HEK‐293T cells were used to construct stable STING‐expressing cell lines. THP‐1 *cGAS*‐KO cells were used to construct cell lines stably expressing cGAS and its mutants. All the cell lines were selected with 3 µg mL^−1^ puromycin twice as previously described.

### 4.7 Generation of Inducible Expressing Cell Lines

cGAS and its mutant‐inducible THP‐1 cell lines were constructed with a lentiviral system. The indicated lentiviral particles were produced by transfecting HEK‐293T cells with cGAS and its mutants‐plenti‐EH‐DEST‐2F or cGAS and its mutants‐plenti‐TRE‐DEST‐2F plasmid together with the pLP1, pLP2, and Plp/VSV‐G vectors. THP‐1 cells expressing Teton‐3G were infected with lentivirus‐containing supernatant to construct the indicated cell lines and selected for puromycin resistance to 3 µg mL^−1^, and polyclonal pools of cells were used for clonal screening and subsequent experiments.

### 4.8 Virus

HSV‐1 was propagated and titrated in Vero cells. Virus titres were measured by means of 50% of the tissue culture's infectious dose (TCID50). The cells were infected with HSV‐1 (MOI = 1) for the indicated time periods.

### 4.9 Antibodies and Reagents

Horseradish peroxidase (HRP)‐anti‐Flag (M2, A8592), anti‐β‐actin (A1978), and Anti‐cGAS (HPA031700) were purchased from Sigma‐Aldrich. Anti‐DNA mouse monoclonal antibody (CBL186) was purchased from Millipore. Anti‐phospho‐STAT1 rabbit monoclonal antibody (Tyr701, 9167), anti‐STAT1 rabbit monoclonal antibody (14994), anti‐phospho‐TBK1 rabbit monoclonal antibody (Ser172, 5483), anti‐TBK1 rabbit monoclonal antibody (Ser172, 3504), anti‐phospho‐IRF3 rabbit monoclonal antibody (Ser396, 4947), and anti‐IRF3 rabbit monoclonal antibody (11904) were purchased from Cell Signaling Technology. Rabbit anti‐alpha‐Tubulin polyclonal antibody (11224‐1‐AP) from Proteintech. Goat anti‐rabbit IgG (H+L) cross‐adsorbed secondary antibody, HRP (A‐16104), goat anti‐mouse IgG (H+L) cross‐adsorbed secondary antibody, HRP (A‐16072) and goat anti‐mouse IgG (H+L) highly cross‐adsorbed secondary antibody, Alexa Fluor 568 (A‐11031) were purchased from Invitrogen.

Protein A agarose (20333), protein G agarose (20399) and NeutrAvidin Agarose Resin (29200) were purchased from Pierce. Anti‐Flag M2 Affinity Gel (A2220), HT‐DNA (438545‐06‐3), DTT (10197777001), imidazole (56750), fluorescent microspheres (L4655), pNPP (N7653) were purchased from Sigma‐Aldrich. Protease inhibitors (11697498001) and phosSTOP Phosphatase Inhibitor Cocktail (4906837001) were purchased from Roche. Hieff Trans Liposomal Transfection Reagent (40802ES03) was purchased from Yeasen. Carboxyfluorescein diacetate (C1031) was purchased from Beyotime. Isopropyl‐beta‐D‐thiogalactopyranoside (IPTG, CA413) and protein marker (DB180‐10) were purchased from MIKX. Neutral red (A600652), 100 bp, 45 bp, 25 bp, 20 bp, 15 bp dsDNA were synthesized by Sangon Biotech. Sequences are listed below:

100 bp dsDNA:

Forward: 5′‐ ACATCTAGTACATGTCTAGTCAGTATCTAGTGATTATCTAGACAT ACATCTAGTACATGTCTAGTCAGTATCTAGTGATTATCTAGACATGGACTCATCC ‐3′;
Reverse:5′‐ GGATGAGTCCATGTCTAGATAATCACTAGATACTGACTAGACATG TACTAGATGTATGTCTAGATAATCACTAGATACTGACTAGACATGTACTAGATGT ‐3′.

45 bp dsDNA:

Forward:
5′‐ AAACAAAAACAAAACAAACAACACAACAAACAAAACAAAAACAAA ‐3′;
Reverse:
5′‐ TTTGTTTTTGTTTTGTTTGTTGTGTTGTTTGTTTTGTTTTTGTTT ‐3′;

25 bp dsDNA:

Forward: 5′‐ AAAACAAACAACACAACAAACAAAA ‐3′;
Reverse: 5′‐ TTTTGTTTGTTGTGTTGTTTGTTTT ‐3′;

20 bp dsDNA:

Forward: 5′‐ AAAACAAACAACAAACAAAA ‐3′;
Reverse: 5′‐ TTTTGTTTGTTGTTTGTTTT ‐3′;

15 bp dsDNA:

Forward: 5′‐ AAACAACACAACAAA ‐3′;
Reverse: 5′‐ TTTGTTGTGTTGTTT ‐3′.

### 4.10 In Vitro Recombinant Protein Expression and Purification

The expression plasmids encoding cGAS and its mutans‐conjugated eGFP tag were transformed into *E. coli* BL21. Freshly transformed cells were grown in LB containing kanamycin (50 µg mL^−1^) to an OD600 of 0.6 at 37°C for approximately 12 h. After induction with 0.5 mM IPTG at 37°C for 8 h, the cells were harvested via centrifugation at 3000 rpm at 4°C for 10 min and resuspended in lysis buffer (50 mM Tris‐HCl pH 7.5, 500 mM NaCl, 20 mM imidazole, 0.035% β‐ME, 5% glycerol, and protease inhibitors). The cells were lysed via sonication on ice and centrifuged (12,000 rpm, 30 min, 4°C) to remove debris. The supernatant was treated with benzonase (Millipore, E1014) to exclude nuclear acid and then purified by incubation with Ni‐NTA agarose beads (QIAGEN, 30210) overnight at 4°C. Ni‐NTA beads were then washed with wash buffer (20 mM imidazole, pH 7.8), and proteins were eluted with elution buffer (50 mM Tris‐HCl, pH 7.5; 500 mM NaCl; 250 mM imidazole; 0.035% β‐ME; 5% glycerol). The purified proteins were further dialyzed by using a PD10 column (GE Healthcare) to exclude high concentrations of salt, followed by concentration with storage buffer (20 mM Tris‐HCl pH 7.5, 300 mM NaCl, 1 M DTT) via Amicon Ultra 30K (Millipore, C134281) at 4°C. The protein concentration was quantified via the BCA method (Pierce, 23250), the 260/280 ratio (under 0.55 to ensure that the nucleic acid was excluded) was verified, and the samples were stored at ‐80°C.

### 4.11 In Vitro PS Assay

For the in vitro PS assay, the purified proteins were mixed at the indicated concentrations with PS buffer (5 mM Tris‐HCl pH 7.5, 200 mM NaCl, and 1 mM DTT) and 5% PEG8000 (Sigma, 89510), followed by incubation with the indicated dsDNA at 37°C. A total of 10 µL of each sample was pipetted onto a glass‐bottom culture dish and imaged via Leica TCS SP8 STED 3X confocal microscopy.

### 4.12 FRAP

The FRAP assay was conducted with a Leica TCS SP8 STED 3X confocal microscope. The fluorescent protein was bleached via a 488 nm laser beam at 100% laser power on a circular region of interest (ROI), and time‐lapse images were collected. The fluorescence intensity was measured and normalized relative to prebleaching time points by a Leica AS Lite. GraphPad Prism was used to plot and analyze the FRAP results.

### 4.13 Luciferase and Reporter Assays

Cells were transfected with plasmids encoding IFNβ‐luc and TK‐luc or ISRE‐luc and TK‐luc, together with plasmids encoding the indicated proteins. After treatment with the indicated stimuli, the cells were then collected with passive lysis buffer (Promega, E1941). Enzyme activity was measured and normalized to the efficiency of transfection based on Renilla luciferase activity levels. The fold induction relative to the basal level was measured in the cells.

### 4.14 Quantification of cGAMP Processing Ability In Vitro

cGAMP processing ability of cGAS in vitro was measured with a Kinase‐Glo Luminescent Kinase Assay (V6071, Promega) according to the manufacturer's instructions.

### 4.15 Quantification of the Abundance of cGAS‐Bound DNA

For cGAS‐bound DNA analysis, we transfected the mCherry plasmid as exogenous DNA into cells two h before cell lysis. Then, the cells were treated with 1% formaldehyde, incubated at room temperature for 10 min, and mixed with 10% glycine from a 1.375 M stock. The cells were washed with ice‐cold PBS 3 times, lysed, and collected with low‐salt lysis buffer (LSB, 50 mM HEPES, 150 mM NaCl, 1 mM EDTA, 10% glycerol, 1.5 mM MgCl_2_, and 1% Triton X‐100). After being sonicated 9 times for 10–20 s at 80% in a sonicator, the extracts were centrifuged at 1000 rpm for 5 min in a cold centrifuge. The supernatants were collected, as cGAS‐bound DNA was coincubated with A+G beads together with IgG or a GFP antibody to pull down WT GFP‐cGAS and its mutants by constant rotation at 4°C overnight. The mixtures were centrifuged, and the beads were washed 5 times with LSB. Two hundred microlitres of elution buffer (50 mM Tris, pH 8.0, 1 mM EDTA, 1% SDS, 50 mM NaHCO_3_) was added to the beads, which were subsequently incubated at 65°C for 10 min. The mixture was centrifuged to collect the supernatant, the beads were eluted again, and the eluates were combined. A total of 21 µL NaCl from a 4 M stock mixture was added to both the input and IP samples, which were incubated at 65°C for 4 h. One microlitre of 10 mg mL^−1^ RNase A was added and incubated at 37°C for 1 h, and 4 µL of 0.5 M EDTA stock solution and 2‐µL of 10 mg mL^−1^ proteinase K were added and incubated at 42°C for 2 h.

The extraction and analysis of cytosolic DNA or cGAS‐bound DNA were performed via a phenol/chloroform/isoamyl alcohol assay, followed by a Quantitative real‐time PCR (qRT‒PCR) assay. The sequences of primers used are as follows:

### 4.16 cGAS Mutation Plasmids Construction

All mutation plasmids were constructed with a Fast Site‐Directed Mutagenesis Kit (TIANGEN, KM101) according to the manufacturer's instructions.

### 4.17 RNA Extraction and Quantitative Real‐Time PCR

Total RNA from whole cells after treating with indicated and appropriate stimulation were extracted by TRIzol reagent (Invitrogen, 10296010), followed by cDNA generation with HiScript III RT SuperMix for qPCR (+ gDNA wiper) (Vazyme, R323‐01). qRT‐PCR was performed in Roche LightCycler 480 System (Basel, Switzerland) by using the 2×PolarSignal SYBR Green mix Taq (MIKX, MKG900–10). Total RNA sample from whole cells were normalized to *RPL13A* expression.

**Table:** 

	Forward	Reverse
*RPL13A*	GCCATCGTGGCTAAACAGGTA	GTTGGTGTTCATCCGCTTGC
*ISG15*	CGCAGATCACCCAGAAGATCG	TTCGTCGCATTTGTCCACCA
*IFIT1*	TCAGGTCAAGGATAGTCTGGAG	AGGTTGTGTATTCCCACACTGTA
*IFIT2*	GGAGGGAGAAAACTCCTTGGA	GGCCAGTAGGTTGCACATTGT

### 4.18 Immunoblotting Analysis

Whole cell lysates (WCL) were collected with LSB buffer after treating with indicated and appropriate stimulation. WCL eluted with 1× SDS Loading Buffer at 100°C followed by SDS‐PAGE experiments. Proteins were transferred to PVDF membranes (Bio‐Rad), blocked with skim milk, and then incubated with the indicated antibodies. Proteins were detected by using Immobilon Western Chemiluminescent HRP Substrate.

### 4.19 Macrophage Phagocytosis, Pinocytosis and Lysosomal Enzyme Activity Test

The phagocytosis of macrophage was tested by the fluorescent microspheres incubated for over 30 min after being treated with indicated and appropriate stimulation, then the phagocytic number was observed by immunofluorescence and counted. Pinocytosis of macrophage was tested by the neutral red, macrophages were incubated with 1% neutral red for 1 h and lysed to detect the absorbance at 540 nm. The lysosomal enzyme activity of macrophage was tested by pNPP, macrophages were incubated with 1 mg mL^−1^ pNPP and 1% Triton X‐100 for 1 h, then using the 3 M NaOH to stop the reaction and detected the absorbance at 405 nm.

### 4.20 RNA Sequencing Analysis

Total RNA was isolated from cells via TRIzol reagent, and sequencing was performed by Annoroad Gene Technology. The fastq data were analyzed with FastQC. All the clean data were mapped to the human genome GRCh38 via HISAT2 v2.0.5 with default parameters [70]. Differential expression analyzes between the two comparison combinations was performed via DESeq2 software. The method of Benjamini and Hochberg was used to adjust the resulting *p* values to control for false discovery rates. Genes with adjusted *p* values ≤0.05 were assigned as differentially expressed genes by DESeq2. GO enrichment analysis and KEGG pathway enrichment statistics of the differentially expressed genes were obtained via clusterProfiler software, and GO terms and KEGG pathways with *p* < 0.05 were considered significantly enriched [71]. The RNA‐seq sequence density profiles were normalized per 10 million reads via bedtools in the R package and visualized via the IGV genome browser.

## Author Contributions

C.Q.Z, S.H.C, L.Z.Z, and H.R.L performed the phase separation experiment and analyzed data. J. W., Z.W., and L.Y.Z. developed PScalpel and PRTLc strategy. J.C., Y.X.W., J.W., and J.Q.L. conceived the study and designed experiments. *J.C*. supervised the study.

## Funding

This work was supported by the National Key R&D Program of China under grant (2020YFA0908700), National Natural Science Foundation of China (82471781, 324B2026, 82341047, 32270922, 32300730, 62073225, 62203134, 62527809, 6247617), National Natural Science Funds for Distinguished Young Scholar (62325307), the Guangdong Province Excellent Youth Team Project (2024B1515040009), Guangdong Basic and Applied Basic Research Foundation (2023B1515120085), Guangdong Science and Technology Department (2023B1212060028), and Fundamental Research Funds for the Central Universities (23yxqntd001), the Natural Science Foundation of Guangdong Province (2023B1515120038), the Shenzhen Science and Technology Innovation Commission (JCYJ20220531103401003, JCYJ20220809141216003, JCYJ20210324093808021, JCYJ20220531102817040), the Guangdong “Pearl River Talent Recruitment Program” (2019ZT08X603), the Guangdong “Pearl River Talent Plan” (2019JC01X235) and the Scientific Instrument Developing Project of Shenzhen University (2023YQ019), Shenzhen Key Laboratory of Media Security Grant (SYSPG2024121117403200).

## Conflicts of Interest

The authors declare no conflicts of interest.

## Code Availability

Our full approach, including data download, preprocessing and modelling, is publicly available from GitHub (https://github.com/zly20020208/PScalpel.git).

## Supporting information




**Supporting file**: advs74353‐sup‐0001‐SuppMat.docx.

## Data Availability

The prediction result data of AlphaFold2 were available from AlphaFold Protein Structure Database (https://alphafold.ebi.ac.uk/). Protein structure data were available from RCSB‐PDB (https://www.rcsb.org/) and CASP14 (https://predictioncenter.org/casp14/). The clinical mutation data were available from ClinVar (https://www.ncbi.nlm.nih.gov/clinvar/). The phase separation data were available from LLPSDB (http://bio‐comp.org.cn/llpsdb/) and PDB (https://www.rcsb.org/pdb/). RNA‐seq data have been submitted to the SRA database under accession number PRJNA1149335. The data that support the findings of this study are available from the corresponding author upon reasonable request.
