# Supplementary material for: Machine Learning‐Guided Engineering of Protein Phase Separation Properties in Immune Regulation
Source: Adv Sci (Weinh). 2026 Feb 11;13(22):e20890. doi: 10.1002/advs.202520890 (PMC13088347; doi:10.1002/advs.202520890)
Supplement: Supplementary file 1 — Supporting file: advs74353‐sup‐0001‐SuppMat.docx. [file ADVS-13-e20890-s001.docx]

Supporting Information

**Machine learning-guided engineering of protein phase separation properties in immune regulation**

***Chenqiu Zhang^1^, Jia Wang^2^, Zhe Wang^2^, Liyan Zhu^2^, Sihui Cai^1^, Luzhi Zhan^1^, Haorui Liang^1^, Yaoxing Wu^3,^****^*^****, Jianqiang Li^4,^****^*^****, Jun Cui^1,^****^*^*

*^1^ MOE Key Laboratory of Gene Function and Regulation, Guangdong Province Key Laboratory of Pharmaceutical Functional Genes, State Key Laboratory of Biocontrol, Innovation Center of the Sixth Affiliated Hospital, Center of Evolutionary Synthetic Biology, School of Life Sciences, Sun Yat-sen University, Guangzhou, Guangdong, China.*

*^2^ College of Computer Science and Software Engineering, Shenzhen University, Shenzhen, China.*

*^3^ Department of Critical Care Medicine, Institute of Precision Medicine, The First Affiliated Hospital of Sun Yat-sen University, School of Life Sciences, Sun Yat-sen University, Guangzhou, Guangdong, China.*

*^4^ School of Artificial Intelligence, National Engineering Laboratory for Big Data System Computing Technology, Shenzhen University, Shenzhen, Guangdong, China.*

*Chenqiu Zhang and Jia Wang contributed equally to this article.*

* Correspondence: wuyaox5@mail.sysu.edu.cn; lijq@szu.edu.cn; or cuij5@mail.sysu.edu.cn

**This file includes:**

Supporting Information text

Supplementary Tables 1 to 4

Supplementary Figures 1 to 9

**Supporting Information text**

**Method**

**BetaFold**

Improvements to the Transformer architecture had been made to enhance its suitability for extracting potential associations between amino acids in protein sequences, while concurrently improving model efficiency. To achieve this, kernel methods were employed to approximate the softmax function, leading to dimensionality reduction during correlation coefficient calculations and subsequent improvement in predictive performance. Additionally, the activation function of the feedforward neural network within the Transformer architecture was modified to ReLU^2^, reducing computational complexity while preserving the validity of higher-order polynomial eigenvectors corresponding to amino acids in the protein sequence. The self-attention mechanism, denoted as Self Attention, expressed as follows


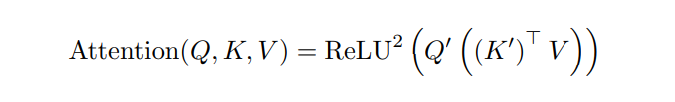


where Q, K, V were the new feature matrices obtained from the input feature matrices through their respective linear transformation functions, Q′ and K′ represented the mappings of Q and K on the kernel function, (·) ⊤ was the transpose operation, ReLU^2^ (·) was the calculation of taking the square of one ReLU function operation. Initially, the feature matrix of the target protein sequence was generated using ProtVec, a variant of Word2Vec specifically designed for biological serialization. This feature matrix was then inputted into a Transformer architecture for Seq2Seq conversion. Through the self-attention mechanism, the feature matrix outputted by the Transformer captures potential associations between amino acids in the sequence. Finally, we paired the output eigenvectors of each amino acid and utilize them as input vectors for a feedforward neural network, serving as a classifier to predict residue contact relationships between amino acid pairs.

In BetaFold module, the feature extraction layer consists of 6 Blocks with d_model=128, multi-head attention heads h=8, and per-head dimension d_k=d_v=16. The application layer is an FNN structure of (128+128)×64 + 64×2. The hyperparameters are set as learning rate=0.001, dropout=0.5, and the optimizer is Adam (Table S3).

**T^3^GCL**

The dataset pertaining to the target task was denoted as A, while the two auxiliary task datasets were represented as B and C, respectively. It was assumed that the auxiliary datasets B and C contain ample well-labeled data, with dataset A being closely associated with the intersection of the two auxiliary datasets. Consequently, despite the absence of labels specifically related to the target task in dataset A, its intrinsically consistent information can still be jointly learned by concurrently training two auxiliary tasks on datasets B and C. To achieve the aforementioned objective, it was imperative to appropriately balance the gradients of the two auxiliary tasks. This was because the learning complexities of the two tasks may significantly differ, resulting in the model primarily capturing the internal consistency features of one task while disregarding the other. In this way, T^3^GCL solved the problem that the traditional method used random data expansion to generate positive pairs in CL, which lead to deviating from the main goal of achieving high sensitivity of the model. Here, we set up 3 datasets A, B and C, which were target dataset, auxiliary dataset and training dataset respectively. The target dataset of phase-separated mutant protein was denoted as A. We used the the clinical mutation dataset NCBI ClinVar as auxiliary dataset B and the phase-separating proteins dataset LLPSDB for C. The appropriateness of this selection hinged upon the existing understanding that anomalous phase separations and transitions bear a causal relationship with various human diseases, including neurodegenerative disorders and cancer.) It was noteworthy that in the dataset B which included cases of the clinical mutation, the original contrastive learning loss function exhibited limited discriminatory ability in distinguishing positive and negative examples. Therefore, we modified the loss function as follows. Denoting the similarity function as sim (x, y) = 1 – arccos (x T y/xy)/π, the loss function was defined as:


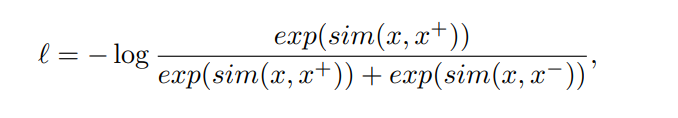


where x was the original protein sequence, x + was the protein sequence with benign mutation, and x^−^ was the protein sequence with pathogenic mutation. Regarding the auxiliary task conducted on dataset C, it was anticipated that the model would acquire the capability to learn the invariance associated with protein phase separation ability. Hence in Formula (3), x and x^+^ represent the sequences of proteins mediating phase separation, and x^−^ denoted the protein sequence without phase separation ability. In order to enhance the learning performance, adjustments were made to the learning rates of the two auxiliary tasks. Specifically, for the gradients of auxiliary task C, denoted as gradC, a modification is applied according to the following equation:


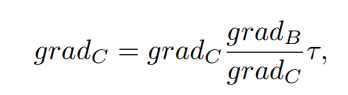


where τ was a proportional constant and was set as 0.05 in this work. Furthermore, different feature extraction techniques were explored to achieve optimal performance. These included self-attention, Graph Isomorphism (GIN), and Graph Attention (GAT). Experimental results demonstrated that the model employing self-attention and GAT yields the most favorable outcomes

This module includes a 100×128 linear transformation layer and a 128×128 feature extraction layer. The hyperparameters are dropout rate=0 and scaling constant τ=0.05 (Table S4).

**PSDM**

To expedite the search for the optimal solution within the vast mutation space, a genetic algorithm was employed. The fitness function for this algorithm was defined as follows:


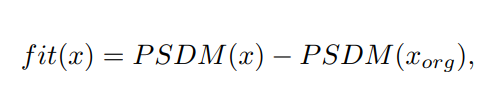


where x represented the mutated protein sequence and x_org_ denotes the original protein sequence. The fitness function quantifies the difference between the phase separation distance matrices of the mutated sequence and the original sequence. The recommendation algorithm followed the subsequent steps. A batch of random mutants for the target protein was generated and utilized as the initial population at first. Subsequently, a selection process was employed to identify and retain a certain number of superior specimens based on the fitness function fit(x). The retained specimens then undergo crossbreeding and mutation to generate a new population with iteratively repeated for a specified number of iterations.

**Table S1.** Dataset of BetaFold

| **Dataset** | **Data Composition** | **Source** |
| --- | --- | --- |
| APSD | Training set (21051)  Validation set (2340) | https://alphafold.ebi.ac.uk/ |
| RCSB-PDB | Test set (731) | https://www.rcsb.org/ |
| CASP14 | Test set (34) | https://predictioncenter.org/casp14/ |

**Table S2.** Dataset of PSDM

| **Dataset** | **Data Composition** | **Source** |
| --- | --- | --- |
| ClinVar | Benign mutation BMP (1590)  Pathogenic mutation PMP (1371) | <https://www.ncbi.nlm.nih.gov/clinvar/> |
| LLPSDB | Mutant protein phase separation (PS^+^) (283)  Native protein phase separation (PS^+^) (493) | http://bio-comp.org.cn/llpsdb/ |
| PDB | Native protein phase separation (PS^-^) (1556) | <https://www.rcsb.org/pdb/> |

**Table S3.** The network structure of BetaFold

| **Feature Extraction Layer** | 100×128 Block | 100×16×8 Self-Attention  100×128 FNN |
| --- | --- | --- |
|  | 128×128 Block | 128×16×8 Self-Attention  128×128 FNN |
|  | 128×128 Block | 128×16×8 Self-Attention  128×128 FNN |
|  | 128×128 Block | 128×16×8 Self-Attention  128×128 FNN |
|  | 128×128 Block | 128×16×8 Self-Attention  128×128 FNN |
|  | 128×128 Block | 128×16×8 Self-Attention  128×128 FNN |
| **Task Head** | (128+128)×64 FNN + BN + *ReLU* ²  64×2 FNN + SoftMax | |

**Table S4.** The network structure of T^3^GCL and FNN

| **Module** | **Network Architecture** | |
| --- | --- | --- |
| T³GCL | 100×128 Linear Transformation Layer  128×128 Feature Extraction Layer | 100×128 Fully Connected Layer  128×128 Self-Attention  128×128 GAT |
| FNN | 128×64 Fully Connected Layer + BN + SeLu  64×16 Fully Connected Layer + BN + SeLu  16×2 Fully Connected Layer + SoftMax | |

**Supplementary Figures**

**
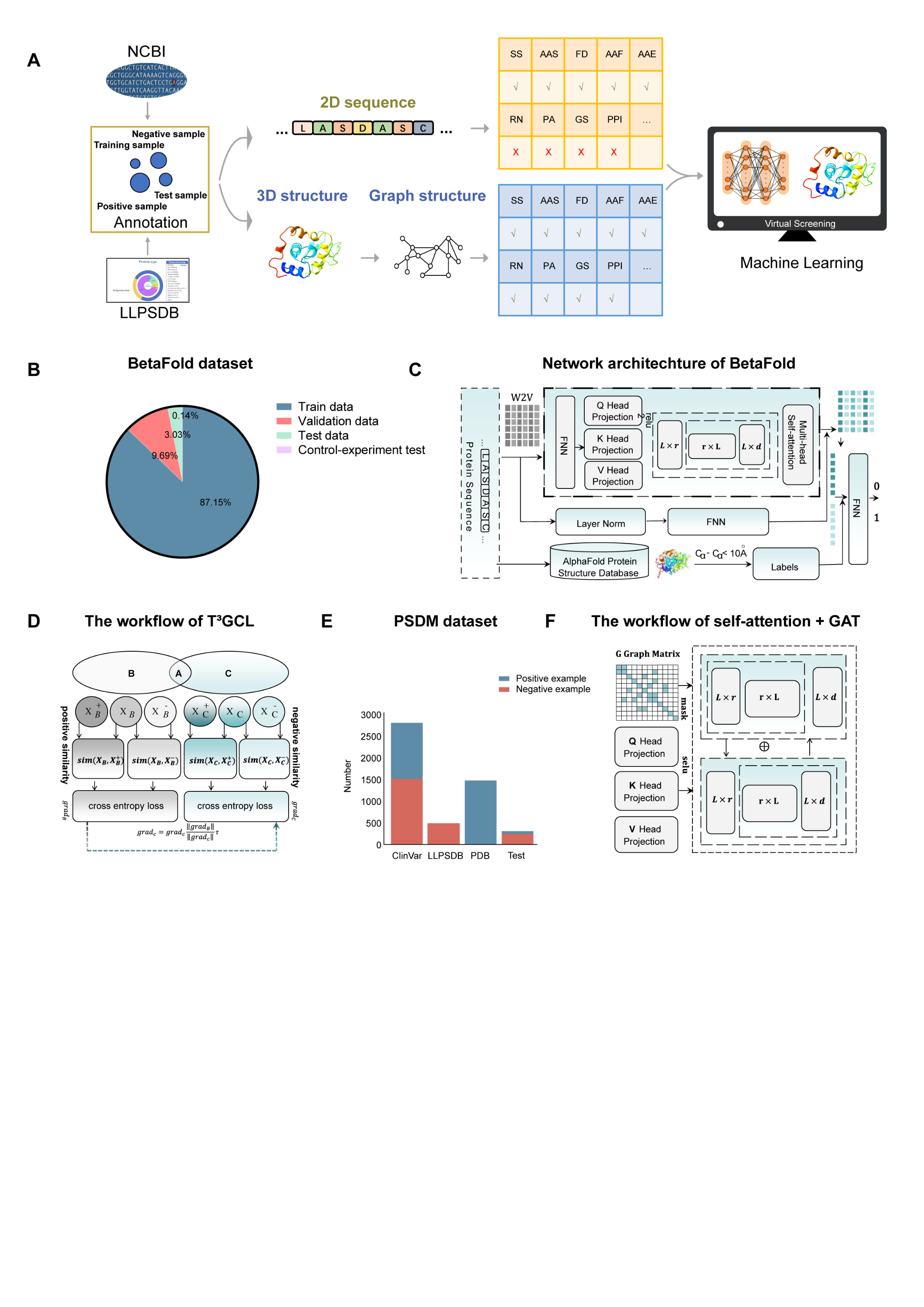
**

**Figure. S1. The workflow of PScalpel framework, related to Figure 1.**

(**A**) Schematic diagram showing the data collection mechanisms of PScalpel. PScalpel was trained by protein 3D structural data which contained more information than protein sequence information. Abbreviation was listed: SS: sequence specificity; AAS: amino acid sequence; FD: functional domain; AAF: amino acid feature; AAE: amino acid electricity; RN: residue node; PA: physical adjacencies; GS: graph structure; PPI: protein-protein interaction.

(**B**) Dataset used in BetaFold for training, validation, test and control experiment.

(**C**) Network architecture of BetaFold. It showed the network architecture of the proposed BetaFold model.

(**D**) The workflow of T^3^GCL.

(**E**) The number of positive and negative example used in PSDM from ClinVar, LLPSDB, PDB and Test set.

(**F**) The workflow of self-attention + GAT. It illustrated the workflow of self-attention + GAT in the PScalpel framework.


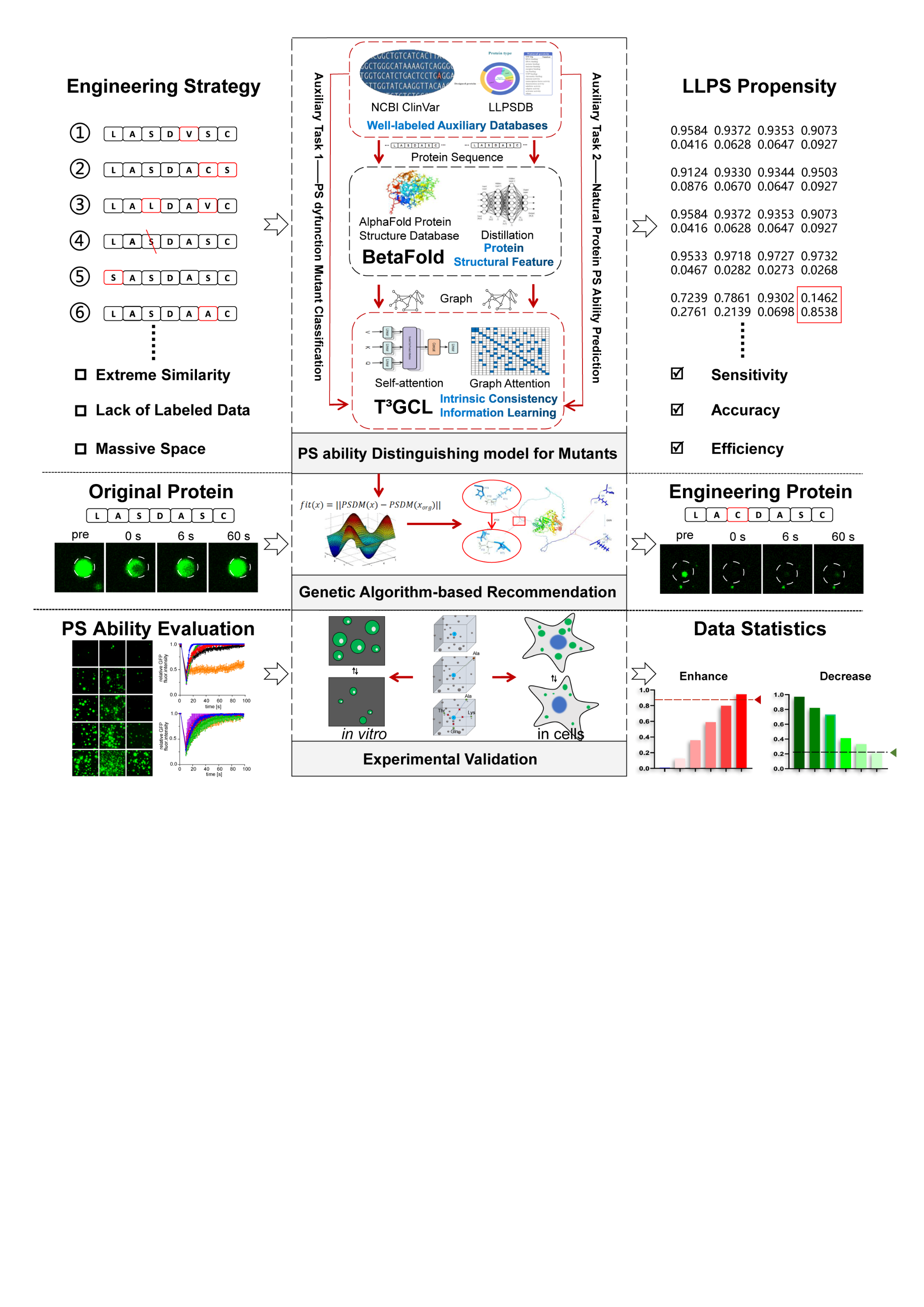


**Figure. S2. Architechure of computational-experimental workflow, related to Figure 1.**

The PScalpel framework operated via a core pipeline of "feature extraction-ability discrimination-mutation recommendation". BetaFold extracted protein structural features, while T³GCL enhanced sensitivity to high-similarity mutants using auxiliary datasets. Together, they formed PSDM, which output quantitative PS ability scores. These scores acted as the GA’s fitness function, enabling the algorithm to iteratively locate optimal mutants in the vast mutation space via selection, crossover, and mutation. PScalpel addressed challenges to realize predictable regulation of protein phase separation. Its predictions were validated through *in vitro* and in cells experiments, with condensate property analyzes yielding expected results. Statistical analysis further identified mutation strategies for subsequent biological engineering or data feedback.


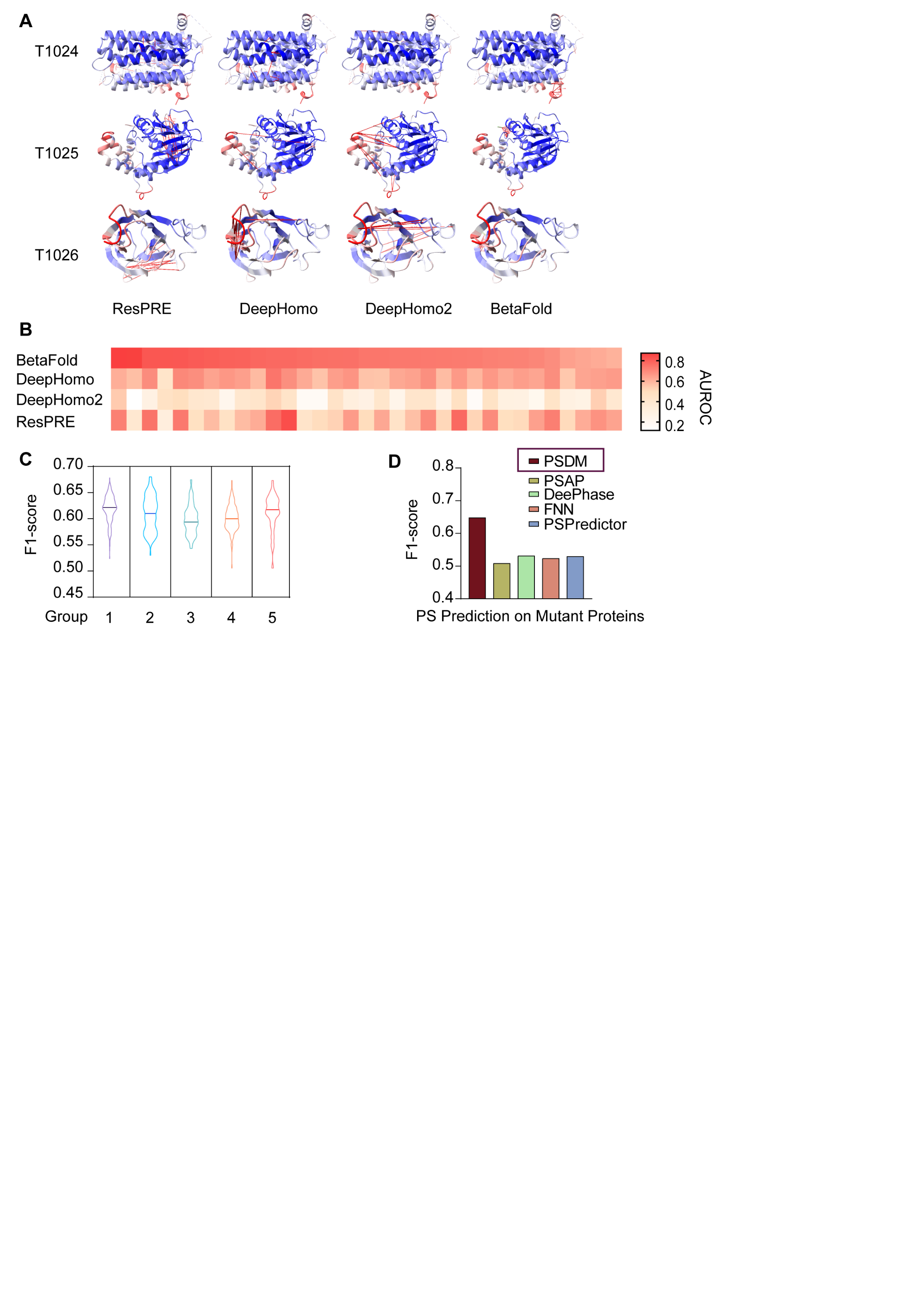


**Figure. S3. Performance of BetaFold and PSDM blocks in predicting protein PS property, related to Figure 2.**

(**A**) The top 10 predicted contacts generated by the ResPRE, DeepHomo, DeepHomo2, and BetaFold models for the T1024 structure. The structure of the realistic target is visually represented by ribbons, where the color scheme distinguishes the regions in pink and blue. The predicted contacts, symbolized by red connections, highlight the residues that are anticipated to exhibit close proximity or interaction according to the respective models' predictions.

(**B**) Comparison of 4 RRCP model on CASP14 dataset with 33 random protein samples. The color of each block corresponds to the AUC value of a predicted protein on the BetaFold model and the corresponding baseline model.

(**C**) The performance of BetaFold robustness, the three-dimensional structural data of natural proteins obtained from experimental calculations were downloaded from the RCSB-PDB database to construct the test dataset.

(**D**) The prediction performance of PSDM on mutant proteins analyzed by F1-score compare with other 4 methods.


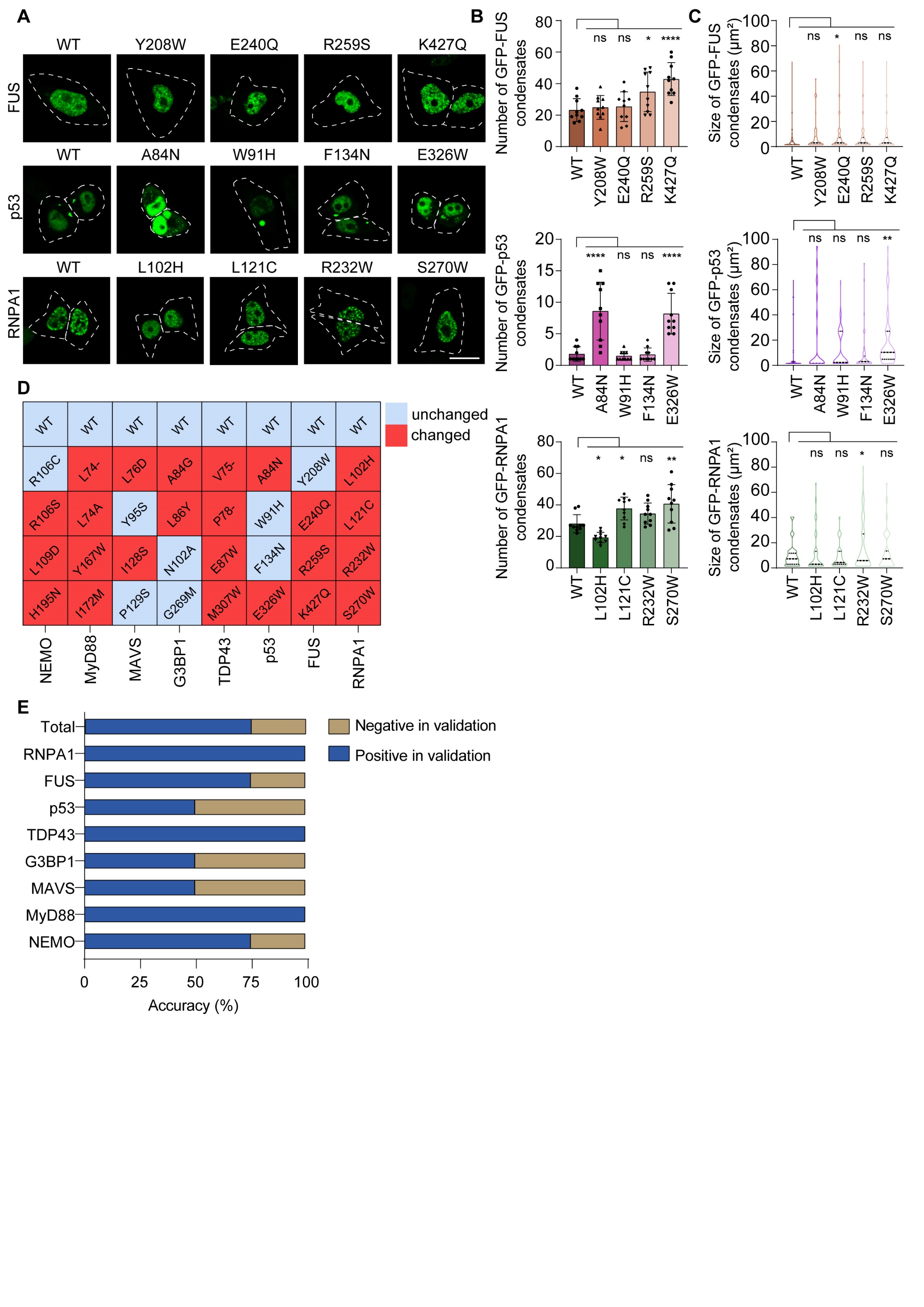


**Figure. S4. PScalpel provides reliable PS ability engineering strategy, related to Figure 3.**

(**A**) Fluorescence microscopy images showed wild type (WT) FUS\p53\RNPA1 and their mutants as labeled. The proteins were overexpressed in HEK-293T cell line with plasmids. The cell boundaries were marked with white dashed lines. Scale bar, 10 μm.

(**B-C**) The number of WT GFP-FUS\p53\RNPA1 and their mutants’ condensates were counted from n = 10 views (B) and the size of condensates was measured from n = 100 condensates (C). Both analyzes were under the condition of proteins overexpressed in HEK-293T cell line with plasmids.

(**D**) Consistency of experiment and prediction results in various proteins.

(**E**) The accuracy of PScalpel prediction under experimental verification.

Data in (**B**) were expressed as mean ± SD of indicated samples for each condition. Data in (**C**) were expressed as median and quartiles of indicated samples for each condition. *P<0.05, **P<0.01, ****P<0.0001, ns, not significant (one-way ANOVA). Similar results were obtained for three independent biological experiments in (**A**).


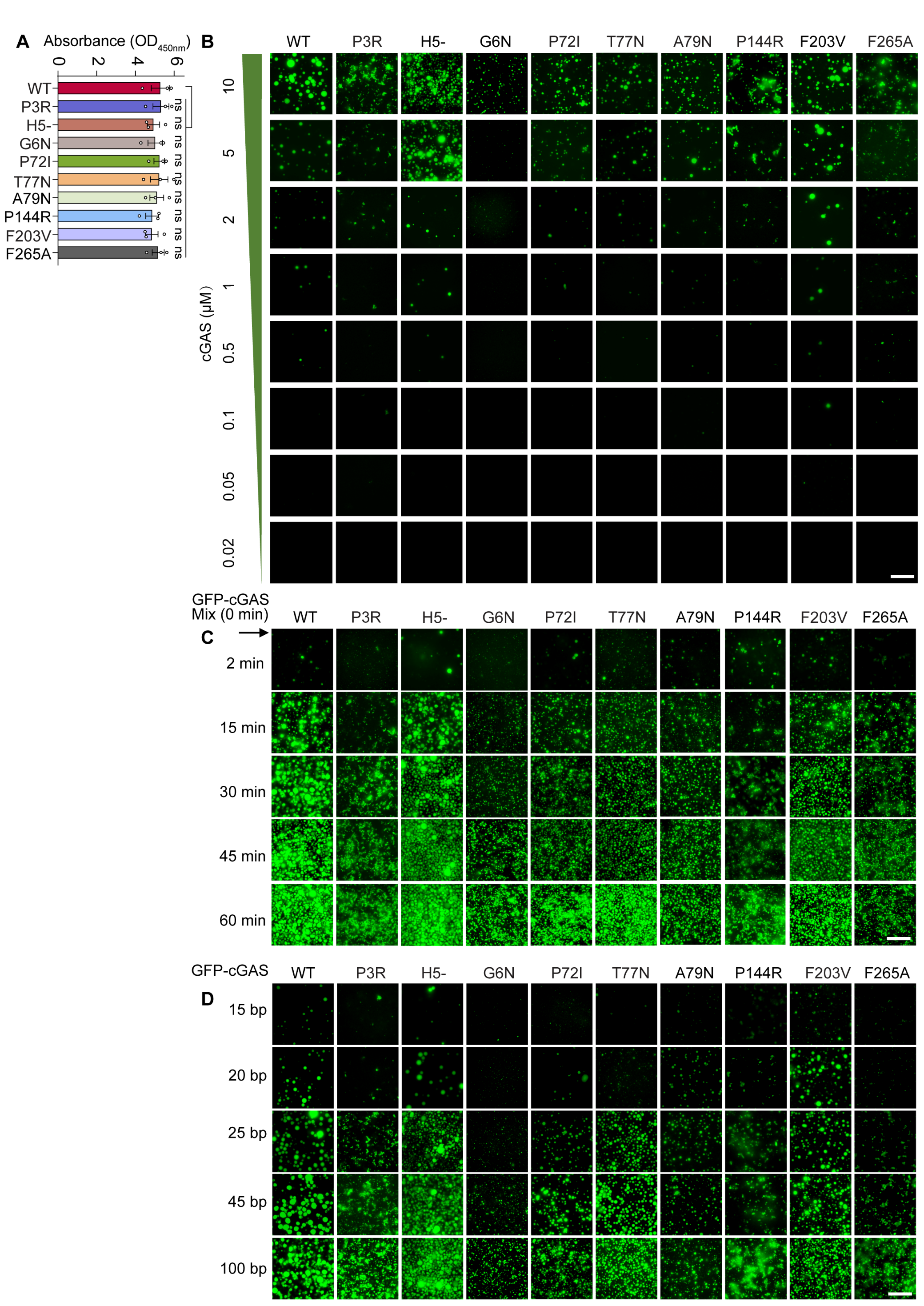


**Figure. S5. PScalpel accurately modulates the PS ability of the immune protein cGAS, related to Figure 5.**

(**A**) Wild type (WT) GFP-cGAS and its mutants recombinant protein were diluted to the same concentration by PS buffer, then tested by BCA assay.

(**B**) Fluorescence microscopy images showed WT cGAS and mutants boundary value. Protein concentrations were used as indicated, incubated with 45 bp dsDNA (2 μg/mL) in PS buffer for 15 min at 37 ℃. Scale bar, 10 μm.

(**C**) Time-lapse imaging of WT cGAS and mutant recombinant proteins mixture with dsDNA phase separation. Condensate formed after mixing of GFP-cGAS (5 μM) with 45 bp dsDNA (2-μg/mL) in PS buffer and matured over 60 min at 37 ℃.

(**D**) Representative images of phase separation by mixing of WT cGAS and mutations recombinant proteins (5 μM) with dsDNA of different lengths (2 μg/mL) in PS buffer for 15 min at 37 ℃. Scale bar, 10 μm.

Data in (**A**) were expressed as mean values ± SD were expressed of n = 3 independent biological experiments. ns, not significant (one-way ANOVA). Similar results were obtained for three independent biological experiments in (**B-D**).


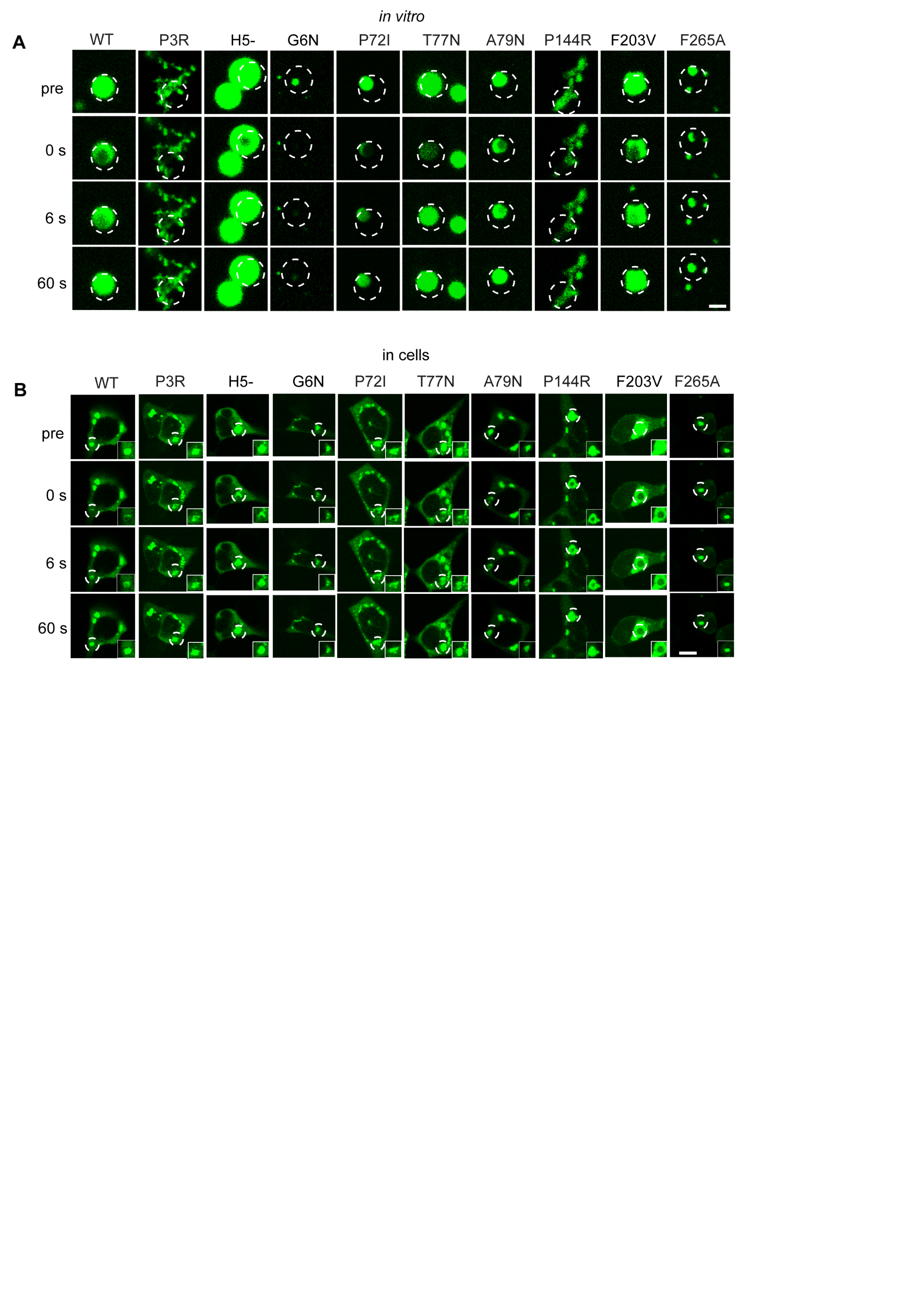


**Figure. S6. cGAS FRAP imaging *in vitro* and in cells, related to Figure 5.**

(**A**) Representative images of confocal microscopy showing recombinant wild type (WT) GFP-cGAS and mutants (5 μM) mixed with 45 bp dsDNA (2 μg/mL) and incubated in PS buffer at 37 °C. Bleaching was performed at the indicated time points and the recovery occurred at 37 °C. Fluorescence intensity analysis of FRAP from n = 6 condensates over 100 s time course. Scale bar, 10 μm.

(**B**) HEK-293T cells expressing WT GFP-cGAS and mutants, transfected with HT-DNA (2-μM) for 12 hours were placed on the dishes at 37 °C. After seeding, bleaching of the cGAS foci was performed and quantification of FRAP of GFP-cGAS condensate was analyzed. The start of recovery after photobleaching was defined as 0 seconds. Representative images of n = 6 cells were shown. Scale bar, 10 μm.

Similar results were obtained for three independent biological experiments in (**A, B**).

**
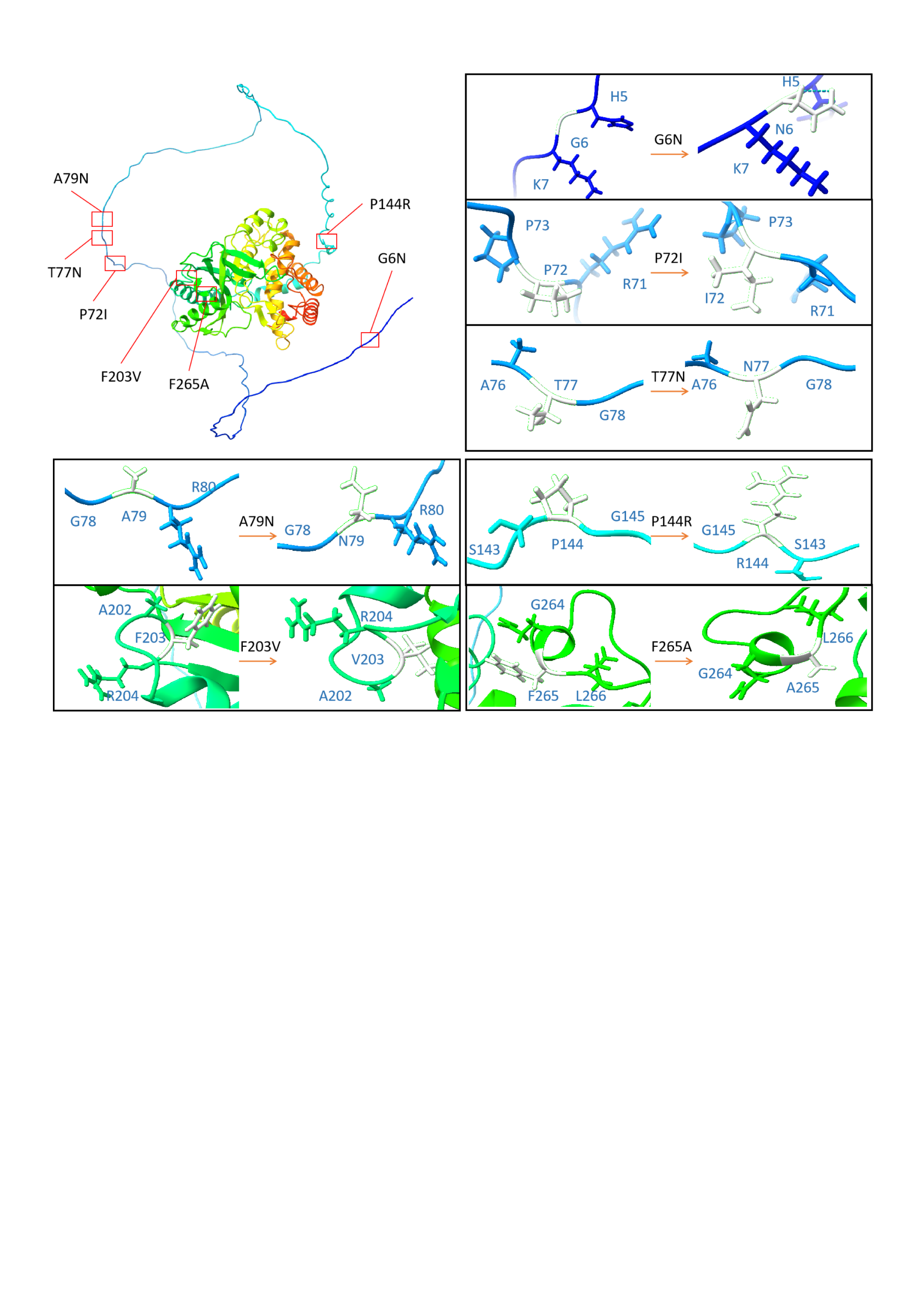
**

**Figure. S7. Specific site mutation of cGAS alters its PS ability, related to Figure 5.**

Visualization of the mutation sites of cGAS predicted by PScalpel before and after. G6N, P72I, T77N, A79N and P144R were located on the N terminal, while F203V and F265A were located on C terminal. Each helix had a different color, with the white part being the mutation site.


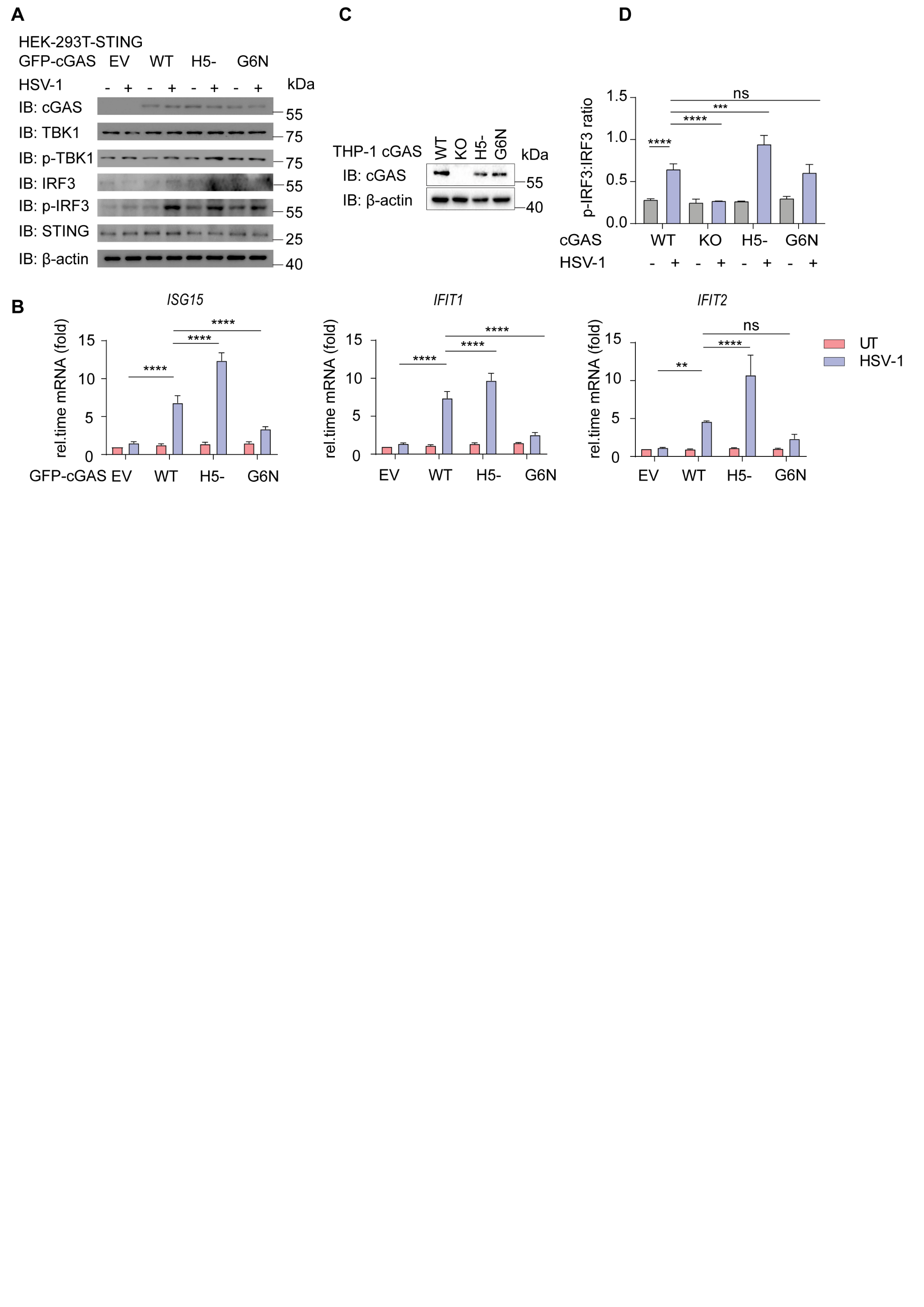


**Figure. S8. PS mutations in cGAS changed its cells’ function, related to Figure 6.**

(**A**) Immunoblot analysis of HEK-293T-STING cells expressing GFP-empty vector (EV), wild type (WT) GFP-cGAS, its H5- or G6N mutants, then treated with HSV-1 (MOI=1) for 24 hours.

(**B**) qRT-PCR with reverse transcription analysis of *ISG15, IFIT1* and *IFIT2* mRNA level of HEK-293T-STING cells expressing WT cGAS or its indicated mutations, then infected with HSV-1 (MOI=1) for 24 hours.

(**C**) Immunoblot analysis of WT, *cGAS* KO, and indicted cGAS mutant expressing THP-1-derived macrophages.

(**D**) Quantification of the expression levels of p-IRF3 shown in Figure 6E.

Data in (**B, D**) were expressed as mean values ± SD were expressed of n = 3 independent biological experiments. **P<0.01, ***P<0.001, ****P<0.0001, ns, not significant (one-way ANOVA). Similar results were obtained for three independent biological experiments in (**A**, **C**).


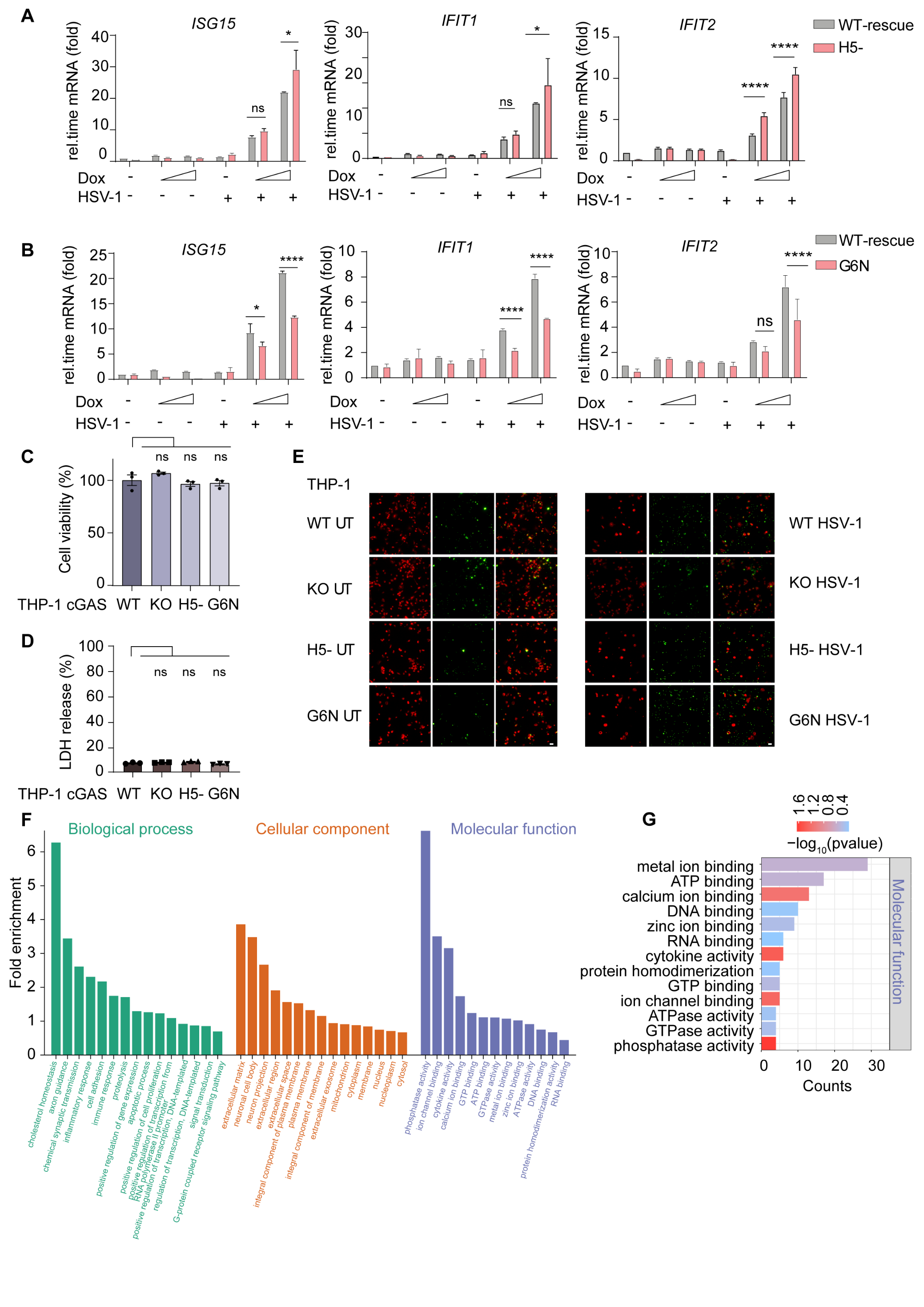


**Figure. S9. cGAS PS mutations precisely regulates distinct downstream responses, related to Figure 7.**

(**A**) qRT-PCR with reverse transcription analysis of *ISG15, IFIT1* and *IFIT2* mRNA level of *cGAS ^wild typ^*^e^ *^(WT)/H5-^* inducible THP-1-derived macrophages treated with doxycycline (Dox, 200 and 400 ng/mL) for 24 hours, then infected with HSV-1 (MOI=1) for 24 hours.

(**B**) qRT-PCR with reverse transcription analysis of *ISG15, IFIT1* and *IFIT2* mRNA level of *cGAS^WT/G6N^* inducible THP-1-derived macrophages treated with doxycycline (Dox, 200 and 400 ng/mL) for 24 hours, then infected with HSV-1 (MOI=1) for 24 hours.

(**C**) The cell viability of THP-1-derived macrophages with the indicated cGAS expression.

(**D**) Lactate dehydrogenase (LDH) release measurement of THP-1-derived macrophages with the indicated cGAS expression.

(**E**) Representative phagocytosis images of THP-1-derived macrophages expressing WT cGAS or its indicated mutations under HSV-1 (MOI=1) infection for 24 hours or left untreated (UT), labeled with carboxyfluorescein diacetate (red), fluorescent microspheres (green). Scale bar, 50-μm.

(**F**) GO analysis of cGAS PS-related genes which trend of gene expression was consistent with that of phase separation ability were arranged by fold enrichment value.

(**G**) Functional annotation of these genes was listed on the right with -log_10_(pvalue) analyzed and arranged by counts.

Data in (**A, B**) were expressed as mean ± SD of indicated samples for each condition. *P<0.05, ****P<0.0001, ns, not significant (one-way ANOVA). Transcriptome sequencing data are mean ± standard deviation of three independent experiments. Similar results were obtained for three independent biological experiments in (**C-E**).
